# A novel approach for the prevention of ionizing radiation-induced bone loss using a designer multifunctional cerium oxide nanozyme

**DOI:** 10.1016/j.bioactmat.2022.09.011

**Published:** 2022-09-21

**Authors:** Fei Wei, Craig J. Neal, Tamil Selvan Sakthivel, Yifei Fu, Mahmoud Omer, Amitava Adhikary, Samuel Ward, Khoa Minh Ta, Samuel Moxon, Marco Molinari, Jackson Asiatico, Michael Kinzel, Sergey N. Yarmolenko, Vee San Cheong, Nina Orlovskaya, Ranajay Ghosh, Sudipta Seal, Melanie Coathup

**Affiliations:** a Biionix Cluster, Department of Internal Medicine, College of Medicine, University of Central Florida, Orlando, FL, USA; b Advanced Materials Processing and Analysis Centre, Nanoscience Technology Center (NSTC), Materials Science and Engineering, College of Medicine, University of Central Florida, Orlando, FL, USA; c CMC Materials, 870 North Commons, Dr, Aurora, IL, USA; d Department of Chemistry, Oakland University, Rochester, MI, MI, USA; e School of Applied Sciences, Department of Chemical Sciences, University of Huddersfield, UK; f Department of Mechanical and Aerospace Engineering, University of Central Florida, Orlando, FL, USA; g Engineering Research Center for Revolutionizing Biomaterials, North Carolina A & T University, Greensboro, NC, USA; h Department of Automatic Control and Systems Engineering, Insigneo Institute for In Silico Medicine, University of Sheffield, Sheffield, S1 3JD, UK

**Keywords:** Ionizing radiation, Osteoporosis, Nanozyme, Cerium oxide, Bone strength, Bone resorption, IR, Ionizing radiation, CeONPs, Cerium oxide nanoparticles, DNA, Deoxyribonucleic acid, ROS, Reactive oxygen species, FDA, Food and Drug Administration, BMSC, Bone marrow derived mesenchymal stem cells, SOD, Superoxide dismutase, CAT, Catalase, Gy, Gray, GPX, Glutathione peroxidase, HIF1α, Hypoxia-inducible factor 1 alpha, SAED, Selected area electron diffraction, XPS, X-ray photoelectron spectroscopy, DFT, Density functional theory, EPR, Electron paramagnetic resonance, ALP, Alkaline phosphatase, COLI, Collagen type I, OCN, Osteocalcin, RANKL, Receptor activator of nuclear factor kappa-Β ligand, MNGC, Multinucleated giant cell, IL-1β, Interleukin 1 beta, IL-6, Interleukin 6, CTSK, Cathepsin K, TRAP, Tartrate-resistant acid phosphatase, CTX-1, Cross-linked C-telopeptide of type I collagen

## Abstract

The disability, mortality and costs due to ionizing radiation (IR)-induced osteoporotic bone fractures are substantial and no effective therapy exists. Ionizing radiation increases cellular oxidative damage, causing an imbalance in bone turnover that is primarily driven *via* heightened activity of the bone-resorbing osteoclast. We demonstrate that rats exposed to sublethal levels of IR develop fragile, osteoporotic bone. At reactive surface sites, cerium ions have the ability to easily undergo redox cycling: drastically adjusting their electronic configurations and versatile catalytic activities. These properties make cerium oxide nanomaterials fascinating. We show that an engineered artificial nanozyme composed of cerium oxide, and designed to possess a higher fraction of trivalent (Ce^3+^) surface sites, mitigates the IR-induced loss in bone area, bone architecture, and strength. These investigations also demonstrate that our nanozyme furnishes several mechanistic avenues of protection and selectively targets highly damaging reactive oxygen species, protecting the rats against IR-induced DNA damage, cellular senescence, and elevated osteoclastic activity *in vitro* and *in vivo*. Further, we reveal that our nanozyme is a previously unreported key regulator of osteoclast formation derived from macrophages while also directly targeting bone progenitor cells, favoring new bone formation despite its exposure to harmful levels of IR *in vitro*. These findings open a new approach for the specific prevention of IR-induced bone loss using synthesis-mediated designer multifunctional nanomaterials.

## Highlights

• At reactive surface sites, cerium ions have the ability to easily undergo redox cycling: drastically adjusting their electronic configurations and versatile catalytic activities.
• A higher fraction of trivalent (Ce3^+^) surface sites, mitigates the IR-induced loss in bone area, bone architecture, and strength.
• A new approach for the specific prevention of IR-induced bone loss using synthesis-mediated designer multifunctional nanomaterials.

## Graphical abstract

**Figure:**
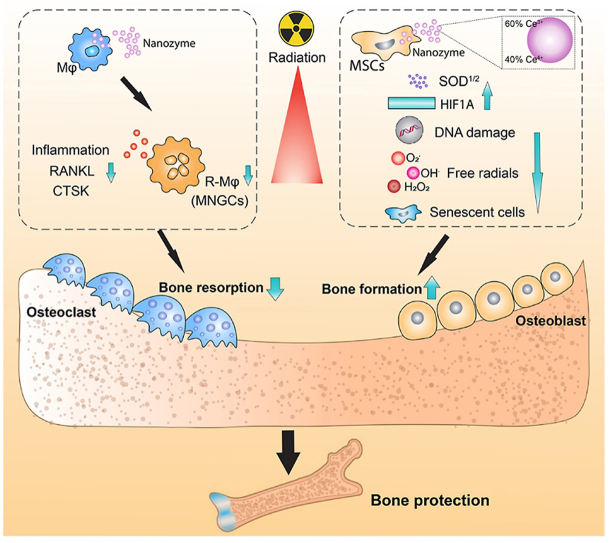

## 1 Introduction

Radiotherapy exposes patients to controlled levels of ionizing radiation (IR) and is an effective and essential component of cancer care and management. Due to its high calcium content, bone tissue is estimated to absorb 30–40% more radiation than other tissues, making it a common site for serious ancillary tissue damage in cancer survivors [1]. Ionizing radiation increases cellular oxidative damage, causing an imbalance in bone turnover that is primarily driven *via* heightened activity of the bone-resorbing osteoclast, resulting in fragile, osteoporotic bone. The risk of suffering an osteoporotic insufficiency fracture is multi-dependent, but can increase by 7.1% [2], 55.7% [3] and 89% [4], due to a variety of factors (*e.g.,* gender, menopausal state, age and comorbidities), exposure parameters (*e.g.,* dose per fraction, total dose) [5], and the cancer itself [6]. Because of IR-induced cell dysfunction, the subsequent bone healing response to fracture is often impaired or absent, resulting in up to 75% of patients unable to self-repair fractures and a further 43% requiring amputation to resolve the complication [7,8]. Although a fundamental and necessary therapeutic tool, the burden of IR-induced damage to healthy bone is a persistent and substantial source of functional impairment, pain and morbidity. The exact pathogenesis of IR-induced bone loss has not been discovered, and despite extensive advances in the research and development for finding nontoxic, safe, and effective prophylactic countermeasures, the FDA has approved only amifostine. Animal models have demonstrated amifostine protects against bone damage [[9], [10], [11], [12]] however, its major drawback is when administered at the high doses required for radioprotection, the drug is toxic, and patients become hypotensive, with both upper and lower GI disturbances [13,14]. This has resulted in frequent adverse clinical events and decreased efficacy [15,16]. Despite subsequent progress made to improve its effectiveness, none of the strategies have resolved the issue of its toxicity/side effects, hence excluding it from general clinical use [17]. Thus, no effective osteopreventative agent exists and patients are instead advised to modify their daily habits to reduce the likelihood of suffering from these complications. Therefore, new insights into IR-induced dysfunction could be of great clinical and therapeutic importance and motivates the continued search for an osteoprotective treatment against bone tissue loss.

The absorption of IR by living cells can directly disrupt atomic structures through the ionization of deoxyribonucleic acid (DNA) and other cellular targets, *i.e.,* causing loss of electrons (oxidation) and simultaneous addition of excess electrons (reduction), with ensuing lesions within the molecular structure [[18], [19], [20], [21]]. This can bring about single and double strand breakage, and tandem lesions including crosslinks to DNA, and functional damage to the cell. Radiation also damages cells *via* the indirect effect of water radiolysis, culminating in the formation of excessive reactive oxygen species (ROS), which are a major concern due to their detrimental cellular effect [22]. The energy deposition to water causes intracellular bursts of ROS, able to microdistribute extracellularly, and far enough to cause a “bystander effect” to cells within the local proximity, damaging critical molecules through protein carbonylation, lipid peroxidation, and enhancing rates of spontaneous gene mutations and neoplastic transformation [20,[23], [24], [25], [26]]. The species produced by the radiolysis of water include radical products (e^-^_aq_, OH^•^, H^•^), and molecular products (H_2_ and H_2_O_2_) as well as protons and hydroxyl ions [27]. In the presence of oxygen, e^-^_aq_ and H-atom (H^•^) radicals are rapidly converted to superoxide anion radical/hydroperoxyl radicals (O_2_^•-^/HO_2_^•^), among other organic radicals that are also formed [28]. It is estimated that about two-thirds of IR damage to DNA in mammalian cells is caused by the hydroxyl radical (OH^•^), as it reacts amply with organic biomolecules found in living organisms [26,29]. The O_2_^•-^ radical is also a major culprit of free radical-mediated toxicity as it is easily generated, and through various pathways or chain reactions, is a primary precursor in the formation of various other harmful ROS [19]. Hydrogen peroxide (H_2_O_2_) is a neutral species believed to have more toxic potential (often ascribed to its kinetic stability and ability to permeate the cell membrane) than O_2_^•–^ and HO_2_^•^ is able to directly generate OH^•^ radicals *via* the Fenton reaction [30,31]. In addition to attacking DNA, ROS initiate the most devastating effect of oxidative stress, which is membrane lipid peroxidation [32]. Thus, both direct and indirect IR-induced dysfunction are initiated *via* a series of molecular and biochemical signalling events that occur during or shortly after IR exposure. These events are responsible for most of the harmful effects of radiation that occur thereafter [23,33].

*In vitro* bone tissue studies demonstrate that human bone marrow-derived mesenchymal stem cells (hBMSCs) to exhibit a dose-dependent decrease in proliferation and a reduced capacity for osteogenic differentiation following exposure to IR [[34], [35], [36]]. Osteoblasts display growth inhibition and reduced bone mineral deposition [37,38]. Macrophages when either directly [39] or indirectly damaged by IR, exert their bystander effect by releasing high concentrations of proinflammatory cytokines that serve to further suppress osteoblastic activity and stimulate osteoclastogenesis and bone resorption [[40], [41], [42]]. The combined dysfunction caused by IR to BMSCs, osteoblasts, immune cells and endothelial cells [43], represents the primary contributor for increased bone loss following exposure to IR [44]. Downregulating excess ROS and DNA damage to cells during and immediately following IR exposure, may limit the subsequent dysfunction and tissue damage observed.

The body possesses an antioxidant defense network including superoxide dismutase (SOD), catalase (CAT), glutathione peroxidase (GPX) and small molecules (*e.g.*, glutathione and vitamins) [22,32,45,46]. The enzymes respectively dismutate O_2_^•-^, and lead to the breakdown of H_2_O_2_. Glutathione provides “chemical repair” *via* H-atom donation to short-lived free radical sites thereby “healing” the biomolecular damage created [29,30,32]. However, following IR exposure, the system becomes overwhelmed [18]. The use of exogenous antioxidants to combat IR-induced damage is not novel (*e.g.,* selenium, and vitamin E [46], amifostine [47] and manganese superoxide dismutase (MnSOD2) [48,49]), but few (*e.g.,* polyphenols, anthocyanins [50]) target bone tissue. However, none are capable of completely preventing IR-induced damage.

The properties that rare earth metals endow, makes them a remarkable strategic resource and thus the focus of this study. The chemistry of rare earth metals differs from other main group metals because of the nature and occupation of the 4f orbitals, which in turn, imparts unique catalytic, magnetic and electronic properties [51]. These unusual properties can be exploited to create new technologies that are not possible with transition and main group metals. Unique nanozymes such as cerium oxide nanoparticles (CeONPs), are a new generation of artificial enzymes that have received much attention because of their exemplary nanozymatic activities, low toxicity and ability to easily and drastically adjust their electronic configurations in response to changes in the bioenvironment [52,53]. These properties are derived from quick and expedient interconversion of the oxidation state between Ce^4+^ (fully reduced) and Ce^3+^ (fully oxidized). The CeONPs feature oxygen vacancies, or defects, in the lattice structure, which arise through loss of oxygen and/or electrons, when alternating between CeO_2_ and CeO_2-x_ during redox reactions. Through this dual and regenerative role as an oxidation and reduction catalyst, many studies have shown that CeONPs possess multiple antioxidant-enzyme-like activities, including SOD, CAT, and peroxidase-like activities. As such, they are expected to scavenge almost all types of noxious reactive species under suitable conditions, outperforming endogenous antioxidants [29,32,51,54] (Supplementary Fig. S1).

Here, we describe a novel approach by which rats originally susceptible to IR-induced bone damage, can become resistant to its toxic effects after treatment with a synthesis-mediated designer multifunctional nanozyme composed of cerium oxide. Most notably, the nanozyme repressed IR-induced inflammation and osteoclastogenesis, and in parallel, liberated osteoblastogenesis *in vitro*, culminating in a bone structure able to maintain its architecture and strength *in vivo*. The mechanistic novelty revealed in this study is based on the heightened presence of Ce^3+^ surface sites, versatile shifts in electronic configuration and the presence of oxygen vacancies (defects) on the nanozyme surface; features that encourage significant overexpression of cytosolic and mitochondrial SOD, while neutralizing increased amounts of ROS (*i.e.,* O_2_^•-^, H_2_O_2_ and OH^•^). Our data also supports the additional mechanistic role of nanozyme-induced hypoxia, which may serve a dual role of reducing ROS formation in conjunction with oxygen extraction, stimulating new bone formation through upregulating hypoxia-inducible factor 1 (HIF1α). Importantly, when increased, both HIF1α and SOD are proteins able to elicit both pro-osteogenic and anti-osteoclastic properties simultaneously, likely critical to the osteoprotective response revealed.

## 2 Results

### 2.1 Synthesis and cellular internalization of two distinct nanozymes

**Fig. 1:**
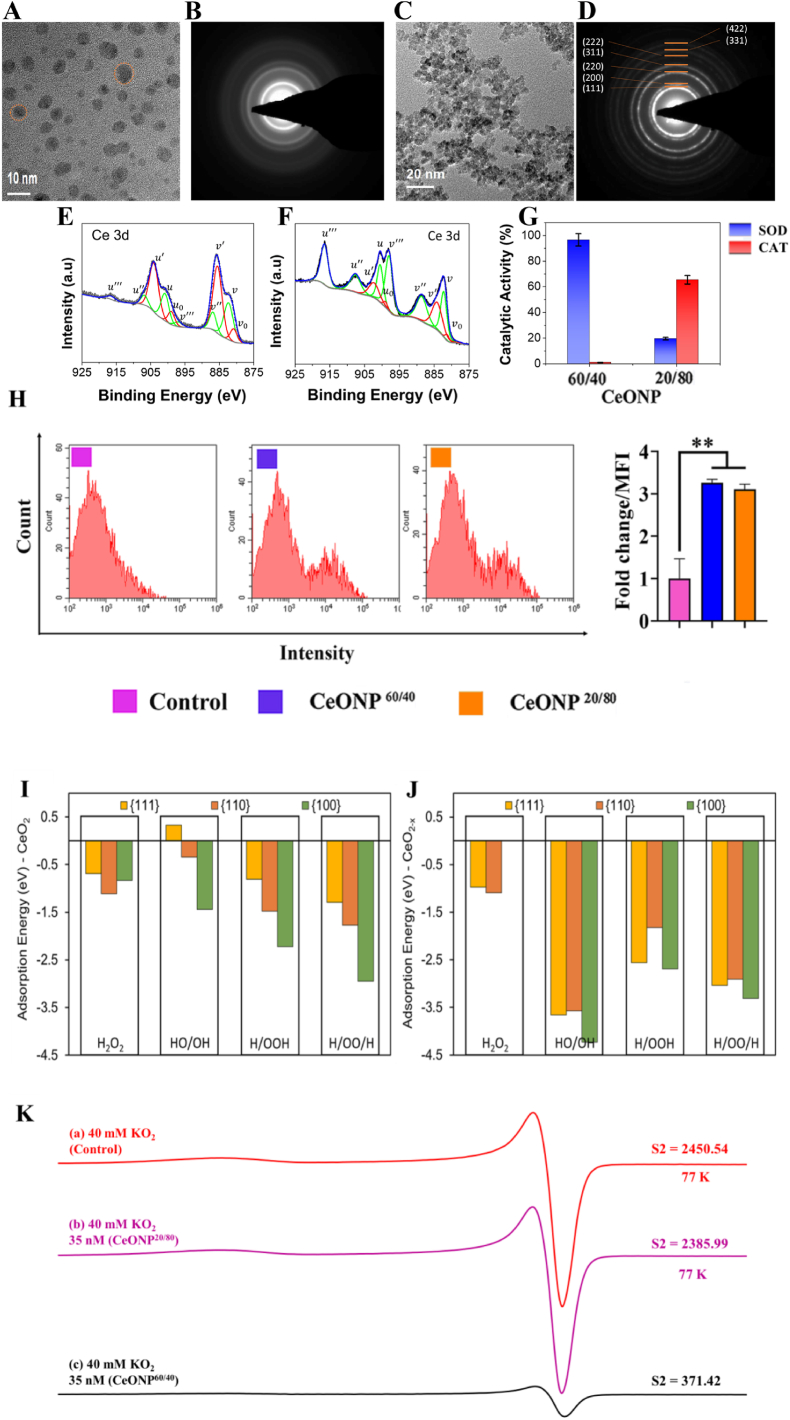
Materials Characterization of CeONP formulations. [A and C] High-resolution transmission electron microscopy (HR-TEM) images of CeONP^60/40^ (a) and CeONP^20/80^ (c) demonstrate spherical (truncated octahedral) morphology of particles in each formulation. Particles from CeONP^60/40^ samples show diameters of 3–5 nm, while those from CeONP^20/80^ are 5–7 nm [B and D] Selected area electron diffraction (SAED) images confirm particle crystallinity for CeONP^60/40^ (b) and CeONP^20/80^ (d) with ring (halo) patterns denoting nanoscale dimensions of primary crystallites and spacing indexed to the cerium oxide crystal structure (observed for both formulations; presented in (d)). [E-F] X-ray photoelectron spectroscopy (XPS) performed over the Ce3d envelope binding energy range allows deconvolution of spin-orbit coupled doublets (3d5/2 and 3d3/2) into multiplet peaks associated with Ce^3+^ (v^0^, v’, u^0^ and u’; red) and Ce^4+^ (v, v”, v”’, u, u”, and u”’; green) redox states. Integration of redox state-specific fitted peak areas allows quantification of Ce^3+^/(Ce^3+^+Ce^4+^) as 20.1% and 61.8% for CeONP^20/80^ (e) and CeONP^40/60^ (f), respectively. [G] Superoxide dismutase (SOD; blue) and catalase (CAT; -red) activities for each formulation are presented (g) with CeONP^60/40^ demonstrating a strong inverse relationship between the tested enzyme-mimetic behavior and CeONP^20/80^ showing a greater CAT activity over SOD (characteristic of respective formulation Ce^3+^ fractions). [H] Flow cytometry analysis showing cellular internalization of FITC-labeled CeONPs in hBMSCs at 24 h: **p < 0.01. [I, J]. The energy of adsorption of ROS evaluated using DFT calculations represents the strength of interactions between the material and the adsorbate. Molecular H_2_O_2_ and its dissociation products (HO/OH, H/OOH, H/OO/H) interact to a lesser extent with the {111}, {110} and {100} surfaces of stoichiometric CeO_2_ [I] than with reduced CeO_2-x_ [J]. [K](a) X-band EPR spectrum of O_2∸_ from a solution of 40 mM KO_2_ in 1:2:2 water/DMSO/IPA after cooling to 77 K for 45 s after KO_2_ dissolution. (b) EPR spectrum of a solution formed identically to that described in (a) with 35 nM CeONP^20/80^. (c) EPR spectrum of a solution formed identically to that described in (a,b) with 35 nM CeONP^60/40^. All spectra were recorded at 77 K. Double integration results are reported as S2 in arbitrary units. The similar S2s of (a,b) indicate that in the presence of CeONP^20/80^, little dismutation of O_2_^∸^ occurred in comparison to the drastically reduced S2 in (c) showing significant SOD-mimetic activity from CeONP^60/40^.

The low formation energies of surface oxygen vacancies are important for oxidation, and the localization of charge as Ce^3+^ state provide power for reduction, altering the electronic configuration, catalytic properties and response to ROS [55,56]. Here, we adjusted the relative fractions of Ce^3+^ and Ce^4+^ surface sites (CeONPs^3+/4+^) respectively, to form two particle formulations (CeONP^60/40^ rich in reduced-state cerium sites, and reduced-state lean CeONP^20/80^); thereby altering the electronic configuration, catalytic properties and response to ROS. Particles from each formulation were observed to be roughly spherical in morphology, suggesting a truncated octahedral morphology with predominately {111} surface facets terminated by {100} facets (Fig. 1[A, C]). Selected area electron diffraction (SAED) images confirm particle crystallinity for CeONP^60/40^ and CeONP^20/80^ with ring (halo) patterns denoting nanoscale dimensions of primary crystallites and spacing indexed to the cerium oxide crystal structure Fig. 1[B, D]). This morphology is characteristic of cerium oxide with a fluorite ($Fm\bar{3}m$, ICSD: 55384) crystal structure, and it is confirmed for both formulations by indexing the selected area electron diffraction patterns. The morphology and average particle size (3–5 nm for CeONP^60/40^ and 5–7 nm for CeONP^20/80^); (Fig. 1[A] and 1[C], respectively) are common to their respective syntheses and were chosen to allow for a more direct determination of the effects from unique cerium formulations and surface chemistry on the tested bio-system [[57], [58], [59]]. The surface (zeta) potential of the CeONP^60/40^ particles was 25.4 ± 0.6 mV with a value of 44.0 ± 7.98 mV measured for the CeONP^20/80^ particles.

X-ray photoelectron spectroscopy (XPS) measurements (Fig. 1[E, F]) were performed to characterize nanomaterial redox state distributions and surface chemistry. High resolution XPS scans over the Ce3d binding energy region allow determination of redox state distribution/fraction between Ce^3+^ and Ce^4+^ [60]. Specifically, spectra are de-convoluted into cerium spin-orbit coupled doublets (3d_5/2_ and 3d_3/2_), with redox-state-specific multiplet peaks further identified for each spin-orbit. The relative amounts from each state were calculated as integrated peak area ratios [Ce^3+^ = A_Ce3+_/ΣA_i_] where A_i_ is the integrated area of the peak “i”. From this calculation, the concentration of Ce^3+^ was found to be 61.8% in CeONP^60/40^ samples (Fig. 1[E]) and 20.1% in CeONP^20/80^ samples (Fig. 1[F]).

Studies noting the approximately inverse relationship between nanoceria formulations’ SOD and CAT activities are well-represented in the literature (35, 41, 42). While the detailed mechanisms informing this relationship remain a subject of current interest [63], the observed dependence on measured Ce^3+^ is well-supported (positive correlation with SOD activity, negative correlation with CAT activity) [58]. A high SOD activity was observed for the CeONP^60/40^ formulation (61.8% Ce^3+^ according to XPS measurements/analysis), in corroboration with earlier published studies (Fig. 1[G]) [58,61,62]. Conversely, CeONP^20/80^ particles show less SOD activity ascribed to lower measured Ce^3+^ (20.1%). In compliment to respective SOD activities, a higher catalase-memetic activity was noted for CeONP^20/80^ samples, whereas negligible catalase-mimetic activity was observed for CeONP^60/40^. As shown in Fig. 1[H], the cellular internalization of FITC-labeled CeONPs into hBMSCs was confirmed using flow cytometry analysis. The mean fluorescence intensity increased significantly after CeONP uptake (*p* < 0.01), indicating internalization into the cell after a 24h period. Both CeONP^60/40^ and CeONP^20/80^ were internalized in similar amounts.

### 2.2 The stabilization of any H_2_O_2_ species is more favorable in the presence of Ce^3+^

Using Density Functional Theory (DFT) calculations, we provide insights into the speciation of CeO_2_ and CeO_2-x_, and unravel the role of Ce^3+^ and Ce^4+^ surface sites on the adsorption behavior of H_2_O_2_ in its molecular and dissociative forms (HO/OH, H/OOH, H/OO/H) (Supplementary Fig. S2). Our results confirm that {111}, {110} and {100} surfaces, species such as OH, OO and OOH are stable forCeO_2_ (Fig. 1[I]) but that the interaction of any H_2_O_2_ species is more favorable in the presence of Ce^3+^ (CeO_2-x_ - Fig. 1[J]). This is particularly noticeable for the products of dissociation of H_2_O_2_ compared to molecular adsorption of H_2_O_2_. Ce^3+^ is therefore a stronger binding and scavenging site compared to Ce^4+^. The presence of Ce^3+^ and associated oxygen vacancy does not allow molecular H_2_O_2_ to be stabilized on {100} surfaces; barrier-less dissociation of molecular species such as water has also been reported [55]. The adsorption process that sees ROS adsorbing directly onto surface oxygen vacancies enables a surface “healing” process too, as surface Ce ions increase their oxygen coordination [64,65] to resemble the stoichiometry of a CeO_2_ stoichiometric surface. The “healing” of oxygen vacancies is also accompanied by a scavenging process whereby the ROS (*i.e.,* OH, OO and OOH) are trapped into surface defect sites with a strong interaction energy as shown in Fig. 1[J].” The scavenging order follows {100} > {110} > {111}. The interaction of dissociated HOO/H and H/OO/H with CeO_2_ and CeO_2-x_ surfaces produce the hydrogen peroxide anion HO_2_^−^ and the peroxide anion O_2_^2−^, respectively adsorbed directly onto the surface, without affecting the surface Ce^3+^/Ce^4+^ ratio. The only exception is seen for the adsorption of H/OO/H onto the {111} CeO_2_ surface, which undergoes reduction (i.e., increasing the Ce^3+^/Ce^4+^ ratio) by forming O_2_ (oxygen molecule) that remains loosely adsorbed onto the surface. Most interesting is the interaction of HO/OH with CeO_2_ and CeO_2-x_ surfaces, which remains the least stable on CeO_2_ surfaces (along with molecular H_2_O_2_) and the most stable on the CeO_2-x_ surfaces. Reduced CeO_2-x_ surfaces undergo an oxidative process and Ce^3+^ is converted into Ce^4+^, thus decreasing the Ce^3+^/Ce^4+^ ratio. On the other hand, because of the inability of stoichiometric CeO_2_ surfaces to oxidize even further (*i.e.,* the intrinsic inability of Ce^4+^ to access higher oxidation states), hydroxyl radicals form onto the surface. Remarkably, we show that excess of ^•^OH on the stochiometric {111} CeO_2_ surface is not stable but that such radicals may be stabilized on the {100} and {110} CeO_2_ surfaces.

### 2.3 Ce^3+^ surface sites selectively neutralize O_2_^•–^

Fig. 1[K] shows a typical O_2_^•–^ Electron Paramagnetic Resonance (EPR) spectrum (***g***_xx_ = ***g***_yy_ = 2.00671, ***g***_zz_ = 2.10196) that has been typically observed in supercooled (frozen) solutions [66,67]. Fig. 1[K] (a) shows the results of the control with no CeONP while Fig. 1[K] (b, c) show the results of solutions formed identically to that of with 35 nM CeONP^20/80^ and CeONP^60/40^, respectively. All spectra are first derivative spectra; thus, double integration of these spectra yields the area under the absorption curve which is reported as S2 in Fig. 1[K] (a-c) in arbitrary units. The similar S2s of Fig. 1[K] (a, b) indicate that little dismutation of O_2_^•–^ (*i.e.,* SOD-mimetic activity) occurred in the presence of CeONP^20/80^ compared to the drastically reduced S2 in Fig. 1[K] (c) which clearly shows strong SOD-mimetic activity from CeONP^60/40^. Thus, these EPR spectral results confirmed that the CeONP^60/40^ particles preferentially catalyse SOD activity and that the higher extent of Ce^3+^ surface sites in CeONP^60/40^ offer specificity for O_2_^•–^ dismutation to a greater degree than those in CeONP^20/80^. The EPR spectra in Figure K demonstrate that the CeONP-mediated dismutation of O_2_^•–^ occurs *via* the following reaction 1:

(1) Ce^3+^ + O_2_^•–^ + 2H^+^ → Ce^4+^ + H_2_O_2_

These results in Figure K also show that Ce^4+^ does not participate in the O_2_^•–^ dismutation and demonstrate little O_2_^•–^ dismutation by CeONP^20/80^. Our density functional theory calculations support that Ce^3+^ rich surfaces would be more effective in O_2_^•-^ scavenging, as all of the reduced surfaces display a higher adsorption energy towards H/OOH and H/OO/H. This also aligns with the mechanism proposed by Perullini et al. [68]. in which SOD mimesis is exclusively due to Ce^3+^ sites on the CeONP surface (see Reaction 1 above).

## 3 *In vitro* analysis of CeONP^60/40^ and CeONP^20/80^

### 3.1 CeONP^60/40^ and CeONP^20/80^ reduce IR-induced intracellular ROS generation and increase mitochondrial O_2_^•-^ scavenging

**Fig. 2:**
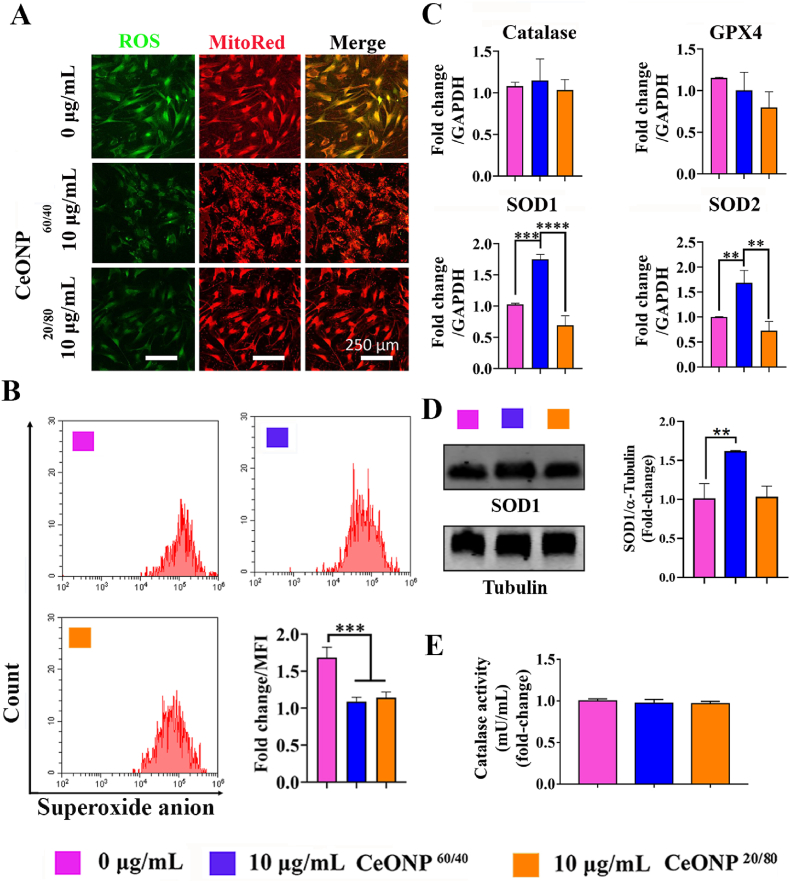
CeONP^60/40^ and CeONP^20/80^ reduce IR-induced intracellular ROS generation, and increase mitochondrial O_2_^•-^ scavenging in primary hBMSCs 24h post-irradiation. The CeONP^60/40^ nanozyme selectively and significantly upregulates *SOD1* and *SOD2* gene expression. [A] Representative confocal microscope images of intracellular ROS and mitochondrial counter-staining in living hBMSCs. After IR-exposure at a dose of 7 Gy, a 160 kV tube voltage, 4 mA tube current, at a distance of 30 cm between the source and the surface (SC 500 smart controller, KIMTRON, USA), cells were stained with ROS/DCFDA (green), and MitoSpy™ Red CMXRos (red). An IR-induced increase in ROS is observed in the untreated control cell group with less expression identified in both groups following 24h pre-treatment with 10 μg/mL of CeONPs and followed by exposure to 7 Gy of irradiation. [B] Flow cytometry results showing a significant decrease in the fold-change of O_2_^•-^ levels after 10 μg/mL of CeONP treatment at 24 h. Similar amounts of mitochondrial O_2_^•-^ scavenging were observed following 10 μg/mL of nanozyme treatment. [C] Expression of antioxidation-related *Catalase, GPX, SOD1 and SOD2* genes after 10 μg/mL CeONP treatment and 7 Gy radiation at 24 h and quantified using qRT-PCR. CeONP^60/40^ selectively and significantly upregulated *SOD1* and *SOD2* expression when compared with CeONP^20/80^ and the control, untreated cells. No other significant differences in gene expression were found. Experiments were carried out in triplicate. All values are given as the mean ± SD. **p < 0.01, ***p < 0.001, ****p < 0.0001. [D] Western blot analysis of SOD1. Endogenous α-Tubulin expression was shown as control. **p < 0.01. [E] Catalase activity was measured using an Amplite® Fluorimetric Catalase Assay Kit.

We assessed both overall ROS and O_2_^•-^ levels in hBMSCs following IR exposure. We have previously reported a significant decrease in ROS following hBMSC pre-treatment with CeONP^60/40^ prior to IR exposure [69] and a similar result was observed in the present study, and as shown in Fig. 2[A]. A reduction in ROS was observed following the supplementation of cells with 10 μg/mL of either CeONP^60/40^ or CeONP^20/80^, confirming their antioxidant properties. The intense red fluorescence stain observed also confirms the presence of metabolically active mitochondria. A reduction in intensity is noted in the cells exposed to radiation without CeONP treatment. ROS generation in the non-irradiated cells was also evaluated, and data shows no significant differences among groups (Supplementary Fig. S3). Flow cytometry analysis further demonstrated that supplementation with either CeONP^60/40^ or CeONP^20/80^, significantly reduced intracellular superoxide anion generation (*p* < 0.001) (Fig. 2[B]).

### 3.2 CeONP^60/40^ increases SOD but not catalase or GPX gene expression in hBMSCs immediately post-IR

**Fig. 3:**
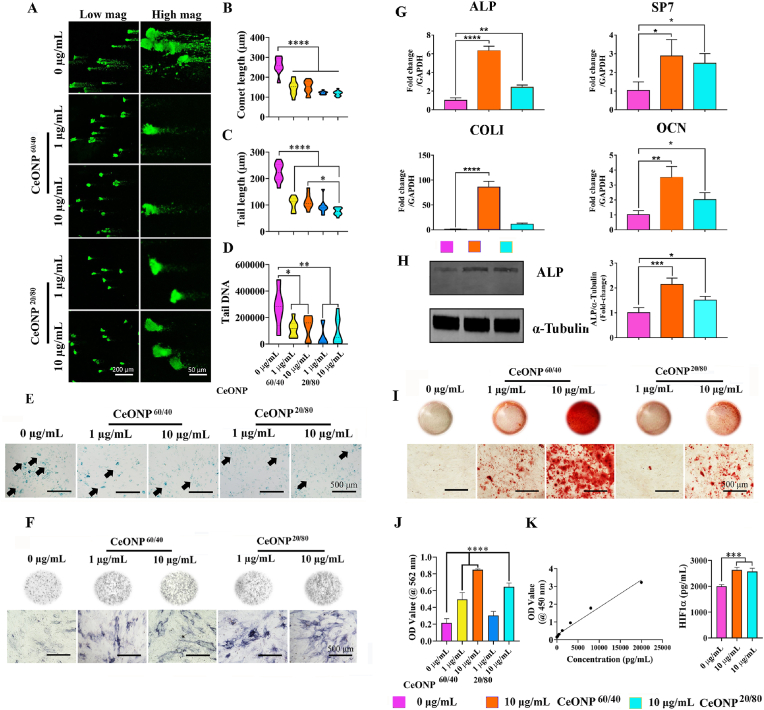
CeONP^60/40^ and CeONP^20/80^ pre-treatment to primary hBMSCs reduces IR-induced DNA damage and cellular senescence. [A] Representative confocal micrographs showing DNA damage 3 days after irradiation and following analysis using the Comet Assay®. hBMSCs were pre-treated with either 1 μg/mL or 10 μg/mL of CeONPs for 24 h prior to a single X-ray exposure to 7 Gy. A significant reduction in DNA damage is observed following treatment with both nanozymes and at both concentrations when compared with the untreated control hBMSCs. Images were captured using confocal laser scanning microscopy. [B] Quantification of comet length: ****p < 0.0001. [C] Quantification of tail length: *p < 0.05, ****p < 0.0001. [D] Quantification of tail DNA: *p < 0.05, **p < 0.01. All values are given as the mean ± SD. [E] Representative micrographs of SA‐β‐gal staining for senescent cells (green) following exposure to IR and with or without CeONP pre-treatment. Using an inverted phase microscope, results show fewer IR-induced senescent cells following pre-treatment with CeONPs at both 1 μg/mL and 10 μg/mL concentrations, and after 28-days of culture. The CeONPs were replenished in the media following irradiation and experiments were carried out in triplicate. Black arrows indicate senescent cells. [F] Representative micrographs following staining for ALP (dark blue) after 14 days of culture in osteogenic media. Qualitative analysis indicates increased levels of ALP in cells treated with 1 and 10 μg/mL of CeONP^60/40^ and CeONP^20/80^ when compared with the untreated control cell group. [G] Expression of osteogenesis-related genes (*ALP, SP7, Col I*, and *OCN*) following X-Ray exposure. *p < 0.05, **p < 0.01, ****p < 0.0001. [H] Western blot analysis of ALP. Endogenous α-Tubulin expression was shown as control. *p < 0.05, ***p < 0.001. [I] The deposition of mineral nodules was qualitatively investigated using Alizarin Red Staining. Areas of red indicate regions of mineralization following 28-days of culture. [J] Mineral deposition by cells was quantified following X-Ray exposure in treated and untreated cells. [K] HIF-1α expression. The level of HIF-1α expression was measured in primary hBMSCs 24h following exposure to 7 Gy. Protein levels were determined using a HIF1α human ELISA kit. The CeONPs were replenished in the media following irradiation. Experiments were carried out in triplicate. All values are given as the mean ± SD. *p < 0.05, **p < 0.01, ***p < 0.001, ****p < 0.0001.

Fig. 2[C] shows that pre-treatment of hBMSCs with CeONP^60/40^ at a concentration of 10 μg/mL followed by IR exposure, resulted in significant upregulation of *SOD1* (*p* < 0.001) and *SOD2* (*p* < 0.01) expression at 24 h, when compared with the control (0 μg/mL) group. No significant difference was found when the CeONP^20/80^ group was compared with control cells and, remarkably, significantly increased levels of both *SOD1* and *SOD2* expression was observed in the CeONP^60/40^ treated cells when compared with the CeONP^20/80^ group (*p* < 0.0001 and *p* < 0.01, respectively). To support this, SOD1 Western blot analysis also indicated increased SOD1 protein expression following 10 μg/mL CeONP^60/40^ treatment (*p* < 0.01) when compared with the 0 μg/mL group (Fig. 3[D]), while catalase activity showed no difference among all tested groups (Fig. 3[E]). In Fig. 1[G], we demonstrated higher SOD activity in CeONP^60/40^ formulation, while CeONP^20/80^ nanozymes show less SOD activity ascribed to lower measured Ce^3+^ (20.1%). At the time point of 24 h, we did not observe significant catalase increase, this may suggest negligible change in peroxide load ascribed either to reduced formation of H_2_O_2_ in response to IR at this time, or the superior scavenging role of the nanozymes *via* conversion of Ce^4+^ surface sites to Ce^3+^.

### 3.3 CeONP^60/40^ and CeONP^20/80^ reduce IR-induced DNA damage in hBMSCs

To determine the radioprotective effect of each nanozyme, hBMSCs were irradiated and DNA damage was quantified using the alkaline Comet Assay. Our results show both formulations, at two doses, significantly reduce DNA damage. As shown in Fig. 3[A, B], the mean comet length of non-CeONP treated cells was 223 ± 35.2 μm, and significantly higher when compared with the 1 μg/mL CeONP^60/40^ (*p* < 0.0001, 105 ± 25 μm), 10 μg/mL CeONP^60/40^ (*p* < 0.0001, 113 ± 27 μm), 1 μg/mL CeONP^20/80^ (*p* < 0.0001, 93 ± 26 μm), and finally, 10 μg/mL CeONP^20/80^ group (*p* < 0.0001, 75 ± 13 μm). The tail length showed a similar trend (Fig. 3[C]). The quantity of tail DNA (Fig. 3[D]) in the non-CeONP treated group, was 273812 ± 153252, which was significantly higher than the 1 μg/mL CeONP^60/40^ (*p* < 0.05, 115745 ± 62682 μm), 10 μg/mL CeONP^60/40^ (*p* < 0.05, 127757 ± 85926 μm), and the 1 μg/mL CeONP^20/80^ (*p* < 0.01, 54489 ± 77552) groups. To control for these results, DNA damage to non-irradiated cells was also evaluated and no DNA damage was measured (Supplementary Fig. S4).

### 3.4 CeONP^60/40^ and CeONP^20/80^ reduce radiation-induced cellular senescence

Cellular senescence occurs naturally as a result of aging as well as through pro-senescence stressors, such as ionizing radiation, resulting in the release of a proinflammatory secretome [70]. Radiation-induced cell senescence was evaluated using β-gal staining. As shown in Fig. 3[E], β-gal-positive staining intensified following exposure to ionizing radiation, however, qualitative observations showed fewer senescent cells in both the 1 μg/mL and 10 μg/mL CeONP^60/40^ and CeONP ^20/80^ groups after 28-days of culture.

### 3.5 CeONP^60/40^ and CeONP^20/80^ do not affect hBMSC proliferation and cytoskeletal morphology following IR-induced damage

Higher or lower levels of ROS can induce delays in different phases of the cell cycle, even in the absence of DNA damage [23]. We evaluated the effect of CeONPs on proliferation and cytoskeletal damage and found no differences between groups at 3- and 5-days post-IR (Supplementary Fig. S5[A-H]). This result was similar to our previous studies (34, 42).

### 3.6 CeONP^60/40^ promotes a greater osteogenic response

Next, we explored the ability of hBMSCs to differentiate into osteoblasts. To compare the protective effect of CeONP^60/40^ and CeONP^20/80^ on irradiation-induced functional damage, hBMSCs were pre-treated with 0, 1 or 10 μg/mL of CeONPs for 24 h before exposure to 7 Gy of irradiation. CeONP treatment within osteogenic differentiation media continued post-IR exposure. By day 7 post-irradiation, and as shown in Fig. 3[F], supplementation of cells with either 1 μg/mL or 10 μg/mL of CeONP^60/40^ or CeONP^20/80^ displayed more intense ALP staining when compared with the 0 μg/mL group, indicating increased osteogenesis in these groups. hBMSCs treated with 10 μg/mL of CeONP^60/40^ resulted in significant upregulation in the expression of *ALP* (*p* < 0.0001), *SP7* (*p* < 0.05), *COLI* (*p* < 0.0001), and *OCN* (*p* < 0.01) when compared with the 0 μg/mL group (Fig. 3[G]). Notably, CeONP^60/40^ significantly increased *ALP* (*p* < 0.0001), *COL1* (*p* < 0.0001), and *OCN* (*p* < 0.05) expression when compared with CeONP^20/80^ treated cells. In addition, Western blot results indicated increased ALP expression in the 10 μg/mL CeONP^60/40^ group (*p* < 0.001) and CeONP^20/80^ group (*p* < 0.05) when compared with the 0 μg/mL group (Fig. 3[H]). Further, CeONP^60/40^ treatment resulted in significantly increased ALP protein release when compared with CeONP^20/80^ treated cells.

On day 28 of culture, Alizarin red S staining was used to evaluate levels of mineralization. Following pre-treatment, irradiation and supplementation with either CeONP^60/40^ or CeONP^20/80^, resulted in an increase in bone mineral deposition in the 1 μg/mL and 10 μg/mL CeONP^60/40^ group, and 10 μg/mL CeONP^20/80+^group when compared with the 0 μg/mL group (Fig. 3[I]). Remarkably, the supplementation of CeONPs^60/40^ at a concentration of 10 μg/mL resulted in a significant >4-fold (*p* < 0.0001) increase in new bone deposition, when compared with the limited mineral deposition formed in the non-CeONP-given irradiated cells (Fig. 3[J]). The level of mineralization in each group was also evaluated in non-irradiated and CeONP-treated cells and results showed the significant effect that all CeONP treated groups had on increasing osteogenesis and bone mineral deposition (Supplementary Fig. S6[A, B]).

### 3.7 Both CeONP^60/40^ and CeONP^20/80^ increase intracellular levels of the hypoxic regulator HIF1α

Next, and to elucidate why CeONP^60/40^ and not CeONP^20/80^ significantly increased bone mineral deposition, we considered whether the increased oxygen extraction activity of Ce^3+^ vacancies may induce transient hypoxia [57]. The hypoxic regulator HIF1α protein is a positive regulator of bone formation [71,72]. Following exposure to IR, our findings show that both formulations of CeONPs significantly increased intracellular levels of the HIF1α protein (*p* < 0.001 in both groups) when compared with control, IR exposed and non-CeONP treated cells. No significant difference was found when the CeONP^60/40^ nanozyme was compared with CeONP^20/80^ (Fig. 3[K]).

### 3.8 CeONP^60/40^ and CeONP^20/80^ do not affect RAW 264.7 proliferation and cytoskeletal morphology following IR-induced damage

Following IR-injury, macrophages are recruited to the irradiated site [73] and when under oxidative stress, the phenotype of the macrophage is key to whether they secrete anti-inflammatory cytokines that promote osteoblastogenesis, or pro-inflammatory cytokines that through RANKL, promote osteoclastogenesis and osteoclastic activity, leading to bone resorption [[40], [41], [42]]. Our previous study demonstrated a reduction in ROS levels within RAW macrophages following CeONP treatment [59]. To investigate the effect of both CeONP^60/40^ and CeONP^20/80^ on RAW 264.7 macrophages, cell proliferation and changes to their actin filaments was investigated on days 1 and 3 following irradiation. However, no significant differences were found between IR only and nanozyme (CeONP^60/40^ and CeONP^20/80^)-treated groups (Supplementary Fig. S7 [A-H]).

### 3.9 CeONP^60/40^ and CeONP^20/80^ repress multinucleated giant cell and osteoclast marker expression following IR-induced cell damage to the macrophage

**Fig. 4:**
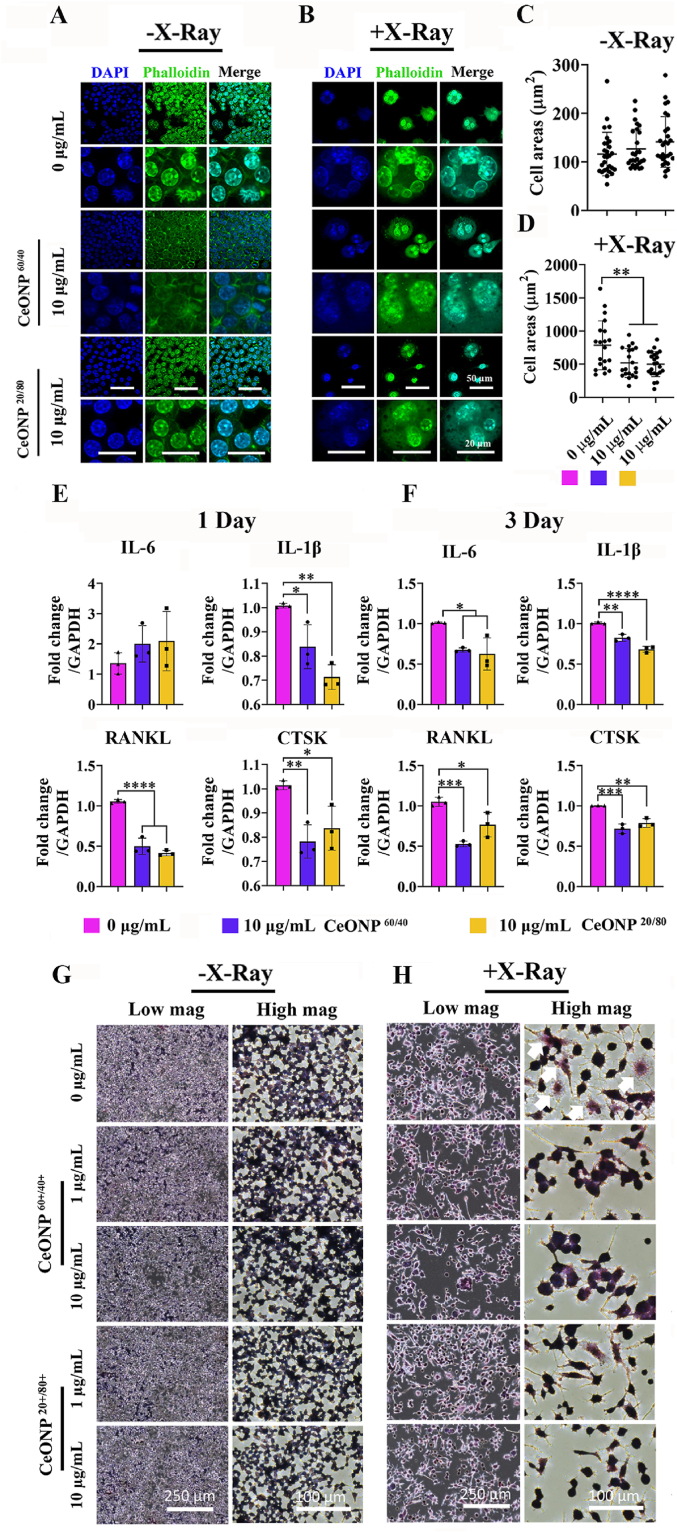
CeONP^60/40^ and CeONP^20/80^ treatment reduces radiation-associated multi-nucleated giant cell formation, pro-inflammatory and pro-osteoclastic gene expression, and osteoclastic activity following X-ray exposure (7 Gy). [A-B] Representative high magnification confocal images of RAW264.7 cells after mock-exposure (A) and X-Ray exposure (B) 3 days post-irradiation and following a dose of 10 μg/mL of CeONPs. [C-D] (results following 1-day exposure are presented in Supplementary Fig. S5). Quantification of cell area (μm^2^) in each group. [E-F] qRT-PCR showing gene expression of pro-inflammatory, and osteoclast markers at 1-day (E) and 3-day (F) post-irradiation. [G-H] TRAP staining of RAW264.7 after mock-X-ray exposure (G) and X-ray exposure (H) 3 days post-irradiation. RAW264.7 pre-treated with either 0, 1 or 10 μg/mL each nanozyme before mock-exposure (G) or exposure (H) to 7 Gy of irradiation. Cells were fixed and stained to detectTRAP activity. White arrows indicate TRAP-positive cells. Experiments were carried out in triplicate. All values are given as the mean ± SD. *p < 0.05, **p < 0.01, ***p < 0.001, ****p < 0.0001.

Radiation-induced multinucleated giant cell (MNGC) formation is a hallmark symbol indicating chronic inflammation; a condition that promotes osteoclastogenesis and increased osteoclastic activity [74]. Our previous study [59] showed CeONP^60/40^ was able to regulate the pro- and anti-inflammatory response and MNGC formation. Here, we assessed the influence of heightened Ce^3+^ activity on the macrophage response, and found that both nanozymes were able to suppress the formation of MNGCs (Fig. 4[A-D] and Supplementary Fig. S8). On day 3 and in the irradiated cell group that received no CeONP treatment, the formation of multinucleated giant-like cells was pronounced. In contrast, fewer of these giant cells were evident in the groups that were supplemented with either 10 μg/mL CeONP^60/40^ or 10 μg/mL CeONP^20/80^. Further quantification of changes in cell area between groups demonstrated significantly larger cells in the 0 μg/mL IR-exposed group when compared with the 10 μg/mL CeONP^60/40^ and 10 μg/mL CeONP^20/80^ groups (*p* = 0.01 in both groups).

Gene expression of the pro-inflammatory (*IL-6, IL-1β*) and osteoclastic markers (*RANKL*, and *CTSK*) were investigated on days 1 and 3 following IR exposure. On day 1, qRT-PCR results demonstrated a significant reduction in *IL-1β* in both the CeONP^60/40^ (*p* < 0.05) and CeONP^20/80^ group (*p* < 0.01) when compared with the control, 0 μg/mL group (Fig. 4[E]). Similarly, a significant reduction in *RANKL* (*p* < 0.0001 in all groups) and *CTSK* (*p* < 0.01 in the CeONP^60/40^ group and *p* < 0.05 in the CeONP^20/80^ group) were also observed when compared with the control, 0 μg/mL group of cells. By day 3, *IL-6*, *IL-1β*, *RANKL*, and *CTSK* were all significantly downregulated in the CeONP-treated groups (*p* < 0.05) (Fig. 4[F]).

To investigate this further, tartrate resistant acid phosphatase (TRAP) staining was used to confirm the formation of active osteoclast-like cells. Macrophages were cultured in the absence of osteoclastic differentiation factors, and as shown in Fig. 4[G-H], irradiation directly induced the formation of “radiation-associated macrophages”, which are characterized by the formation of multinucleated TRAP-positive-like cells with high expression of pro-inflammatory and osteoclast-related markers. On day 3, and following supplementation of cells with either 10 μg/mL CeONP^60/40^ or 10 μg/mL CeONP^20/80^, cells displayed less intensified TRAP staining when compared with 0 μg/mL and IR-exposed group (Fig. 4[H]). These results support previous studies that highlight macrophages as a promising therapeutic target for the prevention or treatment of IR-induced toxicities [41,75].

## 4 *In vivo* analysis of CeONP^60/40^ as a therapeutic following exposure of animals to IR-induced damage

### 4.1 CeONP^60/40^ appears to be a non-toxic nanozyme

**Fig. 5:**
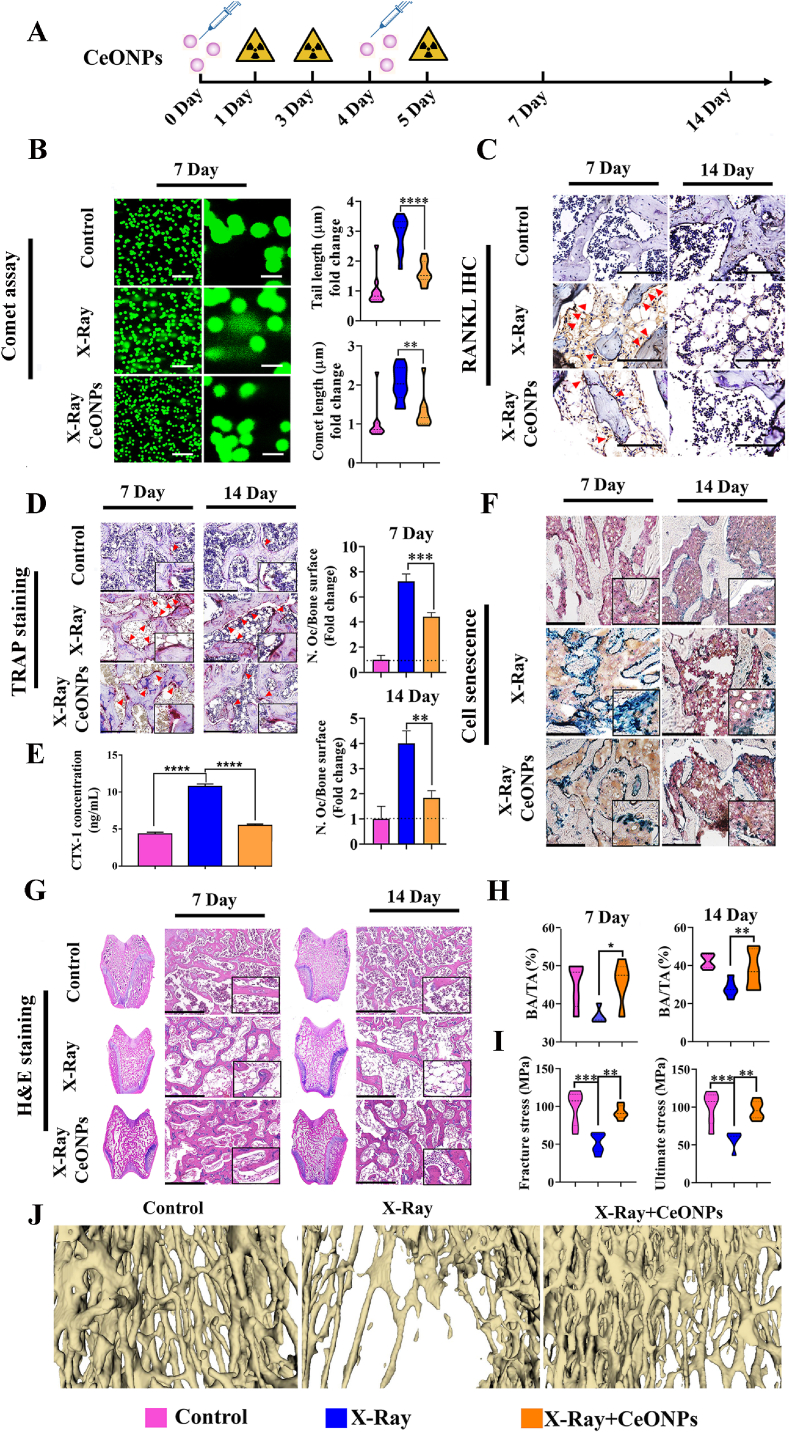
Analysis of the radioprotective effect of CeONP^60/40^ (4 mg/kg) following exposure to levels of radiation able to induce bone loss *in vivo*. [A] Flow chart of the animal experiment. [B] Representative confocal micrographs of DNA damage (Comet Assay®) in bone marrow cells following exposure of the hind limbs to three fractions of 8 Gy (total 24 Gy) on days 1, 3 and 5 (*n* = 6). Quantification of tail length and comet length. Scale bar denotes 250 μm (left panel) and 50 μm (right panel). [C] Representative images of RANKL immunohistochemical staining (red arrows) in each of the experimental groups. Scale bar denotes 125 μm. [D] Multinucleated and active osteoclasts were identified *via* TRAP staining. Red arrows indicate TRAP ^+^ cells. The number of osteoclasts per unit of bone surface (cells/mm) were quantified by bone histomorphometric analyses (2 images/rat, *n* = 6). Scale bar denotes 250 μm. [E] Serum levels of CTX-1 in healthy control animals, X-Ray only, and X-Ray + CeONP^60/40^. ELISA results showed that CTX-1 concentrations were significantly reduced in rats following 4 mg/kg CeONP^60/40^ treatment. All values are given as the mean ± SD. ****p < 0.0001. [F] Representative micrographs of SA-β-gal staining for senescent cells (stained blue). Scale bar denotes 500 μm. [G] Representative images of H&E staining. Note the extensive osteopenia at 14 days post-irradiation in the X-ray group. Scale bar denotes 500 μm. [H] Quantification of trabecular bone area (BA) to total area (TA). *p < 0.05, **p < 0.01. [I] A 3-point bending test was carried out and each tibia loaded to failure at a displacement rate of 0.02 mm/s. Ultimate stress and fracture stress in the control, X-Ray, and X-Ray + CeONP groups are presented (*n* = 6). The tibial parameters measured to obtain stress values are presented in Supplementary Table S1. All values are given as the mean ± SD. **p < 0.01, ***p < 0.001. [J] Representative 3D reconstruction images of the proximal tibia *via* nano-Ct scanning.

Our *in vitro* work showed that the CeONP^60/40^ formulation increased binding of the highly damaging OH^•^ radical, bolstered protective cellular SOD activity and augmented osteoblastogenesis more effectively than CeONP^20/80^. For this reason, we decided to investigate the effect of CeONP^60/40^ in animals and following exposure to 3 fractions of 8 Gy irradiation (total exposure of 24 Gy). A flow chart of the *in vivo* study is shown in Fig. 5[A]. A consideration was whether CeONPs when given at a dose of 4 mg/kg *via* a single tail vein injection twice during the study period, elicits a toxicologic response. Our results showed no histological indications of acute toxicity in the liver, spleen, or kidney 14-days post-treatment. Importantly, a protective response by CeONP^60/40^ was observed in these organs following IR exposure (Supplementary Fig. S9).

### 4.2 CeONP^60/40^ radioprotects cells within the bone marrow niche by reducing DNA damage

Notably, the level of DNA damage to cells within the bone marrow was markedly reduced in the nanozyme-treated rats compared to healthy age- and sex-matched rats (9 weeks old) and following exposure to damaging levels of IR (Fig. 5[B]). IR exposure increased DNA damage to cells and that the mean comet length (*p* < 0.01) and tail length (*p* < 0.0001) in irradiated and CeONP-given animals were significantly lower than the IR only group of animals. These results indicate that injection of the CeONPs markedly reduces IR-induced DNA damage to cells within the bone marrow.

### 4.3 CeONP^60/40^ reduces expression of the osteoclastogenesis factor, RANKL, while also reducing osteoclast number and activity

To investigate changes in the number of active osteoclasts between groups and the role of RANKL, we used immunohistochemical analysis to determine RANKL expression and TRAP staining to measure active osteoclasts from transverse histology sections prepared through the femoral condyle. As shown in Fig. 5[C], the number of RANKL^+^ cells increased in irradiated rats as compared with the non-IR group of rats. In contrast, the expression of RANKL was markedly reduced following 4 mg/kg of CeONP treatment. These results suggest a modulatory role of CeONPs on the osteoclast formation factor, RANKL. Further, and as shown in Fig. 5[D], exposure to irradiation significantly increased the number of TRAP^+^ cells, while no or few TRAP^+^ staining was identified in the non-irradiated group. Remarkably, the number of TRAP^+^ osteoclasts were significantly lower in the rats injected with CeONPs both on days 7 (*p* = 0.001) and 14 (*p* = 0.01) (Fig. 5[D]). The serum level of bone resorption marker, CTX-1, was also significantly reduced following 4 mg/kg of CeONP treatment ((Fig. 5[E]). Our findings demonstrate that CeONPs markedly suppress bone resorption by osteoclasts following exposure to harmful levels of IR.

### 4.4 CeONP^60/40^ reduces irradiation-induced cell senescence

To further clarify whether CeONPs protect bone cells against IR-induced cellular senescence, we analyzed senescence-associated β-gal (SA-β-gal) activity. Immunostaining results indicated that *in vivo* expression of β-gal substantially increased after irradiation and by day 7, minimal β-gal activity was observed in the non-irradiated group of animals, with a more pronounced response in those animals who had been exposed to IR-only. In contrast, animals exposed to IR with no CeONP treatment, showed evidence of β-gal activity. However, β-gal activity was reduced when compared with the IR-only animals, both on days 7 and 14 (Fig. 5[F]).

### 4.5 CeONP^60/40^ reduces bone and trabecular volume loss and increases the mechanical properties of ultimate stress $\sigma_{u}$ and fracture strength $\sigma_{f}$ in cortical bone

Next, we evaluated whether the nanozyme-treated rats were able to maintain their trabecular and cortical bone structure and strength. A transverse section through the center of the femoral condyle was prepared and bone structure was assessed histologically and following H&E staining in samples obtained 7- and 14-days post-irradiation. Rats who underwent irradiation and received no CeONP treatment developed osteoporosis, as indicated by thinner and shorter trabeculae, reduced trabecular connectivity with increased porosity and bone marrow adiposity by day 7, which had further exacerbated by day 14. In contrast, animals receiving both radiation and CeONP therapy, maintained a healthy bone structure and morphology (Fig. 5[G]). Quantification of BA/TA% (Fig. 5[H]) demonstrated a significant reduction in bone area in the non-CeONP treated IR-exposed animals at 7- and 14-days when compared to IR-exposed CeONP-treated animals (*p* < 0.05 and *p* < 0.01 respectively).

Stress-displacement deformation behavior when under 3-point bending test conditions was used to examine the mechanical parameters of ultimate stress $\sigma_{u}$ and fracture stress $\sigma_{f}$ in the tibial mid-shaft of bone in each experimental group. Here, we show the drastic impact that IR has on reducing bone strength, making it more susceptible to insufficiency fracture. Remarkably, and on day-7, each of these bone strength parameters (fracture stress *p* < 0.01, and ultimate stress *p* < 0.01) significantly increased in animals following exposure to irradiation and when given CeONP treatment, compared with the IR-exposed and non-CeONP treated animals. It is important to note that no significant differences were found when animals in the healthy non-irradiated group were compared with those in the IR-exposed, CeONP-treated group (Fig. 5[I]). Load displacement curves, mean fracture and ultimate loads measured and the biomechanical parameters used are presented in Supplementary Fig. S10&S11 and Supplementary Table S1. Additionally, nano-CT analysis demonstrated bone volume loss, while 4 mg/kg of CeONP-treated rats showed reduced bone volume loss when compared to X-Ray group (Fig. 5[J).

## 5 Discussion

The use of CeONPs against radiation-induced damage has been reported in our cell-based osteogenesis study [69] and in other non-bone related studies both *in vitro* [76] and *in vivo* [[77], [78], [79], [80]]. However, the designer tuning of the Ce^3+^/Ce^4+^ ratio towards a specific and protective function, especially in the preservation of osteogenesis following an IR insult *in vivo*, is unknown. Here we reveal the critical contribution of increased Ce^3+^ surface sites in protecting bone against IR-induced damage *in vitro* and *in vivo* and present new insights into the use of a designer artificial nanozyme for the specific prevention of radiation-induced bone loss.

The SOD- and CAT-mimetic activities of CeONPs have not been described here, however, Self et al. [61], and Celardo et al. [52], provide excellent reviews. Our findings support several other studies [52,61,81] and confirm CeONPs designed with a higher fraction of Ce^3+^ surface sites provide enhanced SOD-mimetic activity, while the Ce^4+^ sites dominate catalase-mimetic activity [51,82]. Further, and using EPR, we show that Ce^3+^ surface sites offer specificity for O_2_^•–^ to a greater degree than Ce^4+^ sites. Our findings confirm the role of oxygen vacancies in directly binding H_2_O_2_ and its dissociative forms confirming previous studies [81,83]. However, here we highlight the important and superior participation of Ce^3+^ sites in the adsorption including the increased neutralization of O_2_^•-^, H_2_O_2_ and OH^•^. Our ab initio data also supports a stronger interaction of Ce^3+^ with ROS compared to Ce^4+^.

Our *in vitro* results confirmed that both nanozymes successfully reduced the IR-induced accumulation of ROS within hBMSCs after 24h, and significantly reduced DNA damage and cellular senescence. This indicates the Ce^3+^/Ce^4+^ ratio is mechanistically less relevant here, and through successfully preventing and scavenging ROS, it is conceivable the nanozymes subsequently prevent undesirable perturbations in mitochondrial homeostasis that lead to senescence 28 days later [84]. Interestingly, the DNA tail length in the CeONP^20/80^ group was further reduced when compared with cells supplemented with CeONP^60/40^, suggesting it may confer a superior level of protection. The mechanisms for this remain unclear and as such, warrants future investigation. Importantly, IR-induced DNA damage within the bone marrow niche and cell senescence were both substantially reduced in animals who received CeONP^60/40^ treatment.

Similarly, our findings show the Ce^3+^/Ce^4+^ ratio to be mechanistically less relevant when regulating macrophage activity and upregulating HIF1α expression in hBMSCs following IR exposure. Macrophages play an important role in bone homeostasis and their early infiltration contributes a critical role in IR-induced disease [[85], [86], [87]]. Elevated expression of osteoclastogenic- and inflammation-related factors have been reported in *in vitro* [39] and *in vivo* [[88], [89], [90]] following IR. Notably, CeONPs are able to downregulate inflammatory genes including, IL-1β, IL-6 and TNFα *via* decreased ROS and downregulation of NF-κß [[91], [92], [93]]. Our *in vitro* findings show that IR exposure directly augmented osteoclast-like differentiation in macrophages, as indicated by an increased number of RANKL^+^ and TRAP^+^ cells. Importantly, pre-treatment with the nanozymes significantly inhibited inflammation and osteoclastogenesis-related marker expression while also reducing MNGC formation. The presence of MNGCs has also been suggested to cause chronic inflammation leading to failed osteogenesis and increased osteoclastic differentiation and activity [74]. Increased HIF1*α* is reported to regulate inflammation and polarize macrophages towards the pro-healing M2 phenotype *via* NF‐κß [[94], [95], [96], [97], [98]]. Further, HIF1α is a positive regulator of bone formation, increasing osteoblastogenesis, enhancing bone defect healing *in vivo* [[99], [100], [101]], while selectively downregulating osteoclastogenesis [[102], [103], [104]], thus favouring bone deposition. Our findings reveal both nanozymes significantly upregulated HIF1α 24 h following exposure to IR. Das and colleagues [57] demonstrated that CeONPs activate *HIF1α* through modulation of intracellular O_2_ levels; first extracting O_2_ and then liberating it in the catalytic cycle: CeO_2-x_ + 0.5xO_2_ ⇆ CeO_2_. Thus, increased levels of HIF1α may offer a role in reducing inflammation while simultaneously promoting osteogenesis and repressing osteoclastogenesis. This was validated in part, by a reduction in RANKL^+^ and TRAP^+^ cells in our *in vivo* IR model. The direct effect of CeONPs on osteoclastic behavior is emerging. Through scavenging or generating ROS and *via* NF-κß, a study by Yuan et al. [105] reported CeONPs facilitated osteoclast formation at lower concentrations, but inhibited osteoclastogenesis at higher concentrations *in vitro*. In contrast, CeONPs have also been reported to elicit no effect on osteoclast activity *in vivo* [106]. While our findings support a significant reduction in both TRAP^+^ active osteoclasts on the bone surface, and CTX-1 protein levels with serum following IR, and nanozyme treatment *in vivo*, this study did not investigate the direct response to osteoclasts. Future studies will seek to further clarify its role in regulating osteoclast function and activity.

Further *in vitro* analysis revealed a disparity in the efficacy of the two nanozymes. An emerging protective mechanism observed in our study, was that CeONP^60/40^ exclusively and significantly increased expression of both cytosolic *SOD1* (Cu/Zn-SOD) and mitochondrial *SOD2* (Mn-SOD) in hBMSCs 24h following IR exposure. SOD is indicated in preventing apoptosis and precancerous cell changes, maintaining mitochondrial integrity, protecting enzymes, membranes, microsomes, DNA [49,[107], [108], [109], [110], [111]] and in reducing IL-1β and TNFα production [49]. Notably, protecting lung tissue [112,113], the oral cavity [114,115] and the esophagus [116,117] from IR-induced damage. Both SOD1 and SOD2 have been suggested as therapeutic targets for bone disorders. *Sod1*-deficient mice exhibit reduced enzymatic collagen cross-linking, low bone turnover and develop significant bone fragility [118]. Increased levels of SOD2 is vital for bone metabolism and in suppressing ROS and thus osteoclastic differentiation [[119], [120], [121]]. SOD upregulation may inhibit RANKL-induced osteoclastogenesis, while simultaneously enhancing osteogenesis [76,122]. While our findings serve to further support its protective role, the mechanism of heightened Ce^3+^-SOD related activity remains undiscovered. SOD is a metalloenzyme and requires a redox active transition metal in the active site for activity against O_2_^•-^. SOD proteins have been shown to bypass the dismutation cycle and use external redox equivalents (*i.e.,* alternative metal species) [123]. However, it is important to mention that metal ion promiscuity is highly irregular. Thus, we cautiously speculate that CeONPs may provide an available, alternative, and facile redox equivalent and that the Ce^3+^ sites offers greater selectivity and binding affinity to both SOD1 and SOD2 or confers higher stability or a more beneficial charge transfer efficiency, potentially affording greater reaction kinetics [124].

Importantly, we also show more CeONP^60/40^-induced mineralized nodule formation compared to CeONP^20/80^ *in vitro*. To support this, our data confirmed a significant increase in *ALP*, *COL1*, and *OCN* gene expression, and significantly increased ALP protein expression in response to supplementation with CeONP^60/40^ compared with CeONP^20/80^. The role of CeONPs in upregulating osteogenic protein expression during differentiation has been previously reported [69,125]. Pathway analysis by Luo and colleagues [126], showed that CeONPs enhanced the nuclear translocation of β-catenin and activated the canonical Wnt pathway by promoting sequence similarity 53 member B/simplet expression. This supports our findings as the Wnt/β-catenin signaling pathway regulates osteogenic differentiation, and bone formation, and Wnt pathway activation contributes to increased expression of downstream osteoblast-related genes including, *OCN, ALP* and *COL1* [127]. Further to this, our findings reveal the importance of increased Ce^3+^ surface sites in modulating cell fate and increasing bone deposition *in vitro*. Interestingly, our results are in contrast to Li et al. [128], where a CeONP coating was applied to a titanium surface using magnetron sputtering. The authors report the increased osteogenic differentiation of rat hMSCs *in vitro* and bone formation *in vivo* when a higher Ce^4+^, and not Ce^3+^ fraction, was investigated. It was speculated this balance in Ce valence may have delivered a system more effective in scavenging ROS, thereby promoting osteogenic differentiation and bone formation. However, here it is important to consider that IR exposure induced a significant increase in highly damaging O_2_^•–^, H_2_O_2_ and OH^•^, and our data suggests that when under these physiological conditions, an increased fraction of Ce^3+^ surface sites may be critical in selectively scavenging these ROS, potentially restoring redox balance, and healthy cell function. Nevertheless, and taken together, these findings indicate that manipulation of the Ce^3+^/Ce^4+^ ratio can modulate bone formation and that further research will be needed to identify the exact mechanism/s involved.

Our *in vivo* work confirmed that CeONP^60/40^ given *i.v* to rats significantly reduced IR-impaired architectural decline and bone tissue loss and that non-CeONP treated rats developed fragile, osteoporotic bone. Remarkably, animals who received the nanozyme prior and during IR-exposure, demonstrated a significantly higher mean fracture stress and ultimate stress, with values similar to the non-irradiated, control animals. Although bone area was maintained in CeONP-given, and IR-exposed animals, differences in bone formation rates *in vivo* were not investigated. Therefore, any nanozyme-induced increase in new bone formation was not assessed in this study. However, our findings indicate that the nanozyme is able to deliver a radioprotective effect and the heightened IR-induced susceptibility to insufficiency fracture was mitigated in this model.

**Fig. 6:**
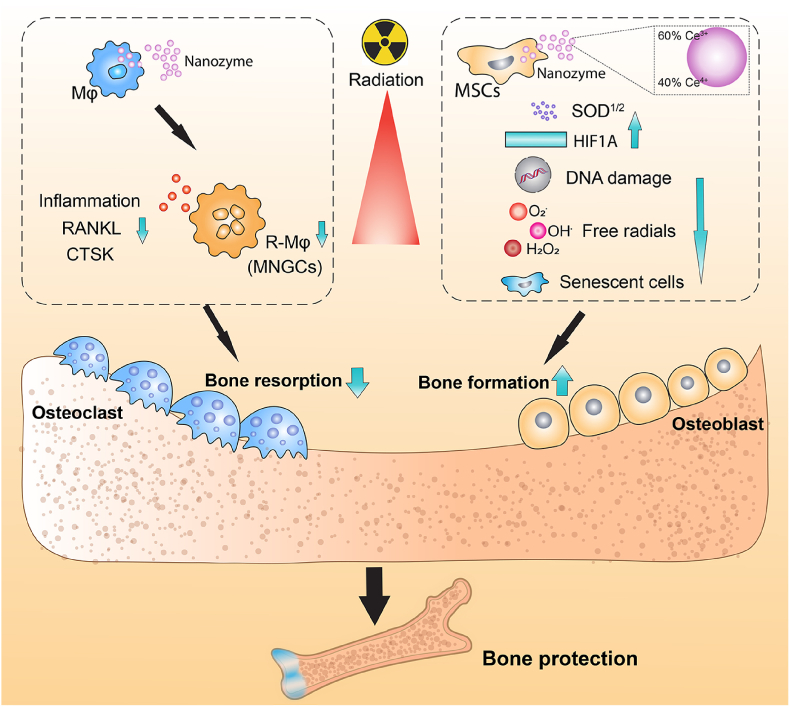
Schematic diagram highlighting the mechanistic avenues of protection provided by CeONPs against irradiation-induced bone loss. Radiation polarizes macrophages into radiation-associated macrophages (R-Mφ), the formation of multinucleated giant cells (MNGCs), with high expression of osteoclastogenesis- and inflammation-related markers and osteoclast activity. These were all significantly repressed following CeONP treatment *in vitro*. Further, CeONP treatment neutralized the highly damaging O_2_^•–^, H_2_O_2_ and OH^•^, decreased DNA damage, increased bone-promoting (and anti-osteoclast) HIF1α protein levels, increased anti-inflammatory, pro-osteogenic and anti-osteoclastogenesis SOD expression, bone mineral deposition and reduced cell senescence thereby liberating osteoblastogenesis *in vitro* and significantly protecting bone against IR-induced bone fracture *in vivo*. The nanozyme designed to possess an increased relative fraction of Ce^3+^ surface sites, provided superior protection *in vitro.* This may be due to the enhanced SOD-mimetic activity, higher adsorption towards H/OOH and H/OO/H, an increase in interaction with ROS on the predominant {111} surfaces, as well as the selective and significant increase in both cytosol and mitochondrial *SOD1* and *2* gene expression.

In conclusion, we have demonstrated that rats treated with a nanozyme results in bone tissue able to maintain its architecture, volume and strength *in vivo,* despite exposure to harmful levels of IR. Analyses revealed the nanozymes were multifaceted and able to prevent intracellular ROS accumulation, DNA damage, senescence, reduce pro-inflammatory and pro-osteoclastogenic markers while increasing pro-osteogenic HIF1α protein release *in vitro*. However, the nanozyme possessing increased Ce^3+^ sites provided important and superior protection through the presence of increased oxygen vacancies leading to stronger interaction with ROS and increased neutralization of O_2_^•-^, H_2_O_2_ and OH^•^. We also reveal Ce^3+^ exclusively targeted pro-osteogenic cytosolic and mitochondrial *SOD* overexpression. Overall, CeONPs, may represent a novel multifunctional therapeutic strategy for mitigating IR-induced damage (Fig. 6), and nanomaterials with a further increased trivalent fraction, may also have a therapeutic schema for IR-induced osteoporosis.

## 6 Methods

### 6.1 Generation of tuned biomimetic nanozymes

#### 6.1.1 Synthesis of a nanozyme designed for greater relative SOD activity (CeONP^60/40^)

Synthesis was performed based on a previously published protocol [129]. Cerium (III) nitrate hexahydrate (99.999% purity; Sigma Aldrich) was added to 50 mL of deionized water and allowed to dissolve completely. The Ce^3+^ ions were converted to a highly hydrated, cerium (IV) oxide through addition of 3% hydrogen peroxide to a pH below 3.5 and a final cerium concentration of 5 mM. From here, the solutions were left standing away from light and aged for up to 8 weeks. Aging was performed to allow degradation of excess hydrogen peroxide, by catalytic surface reactions, and for equilibration of particle phase composition/surface character (aging effects).

#### 6.1.2 Synthesis of a nanozyme designed for greater relative CAT activity (CeONP^20/80^)

A forced hydrolysis technique was used to generate nanoparticles tuned to possess a lower fraction of Ce^3+^ surface sites, relative to Ce^4+^. Synthesis was performed based on a previously published protocol [57]. Specifically, 1.24 g of cerium (III) nitrate hexahydrate (99.999% purity; Sigma Aldrich) was stirred in 50 mL of water for 1 h followed by titration with 30% ammonium hydroxide (ACS grade, Alfa Aesar) to force precipitation of nanocrystalline cerium (hydro-)oxide over 4 h of stirring. The solution was then centrifuged at 10,000 rpm to collect the sedimented particles and the sediment was washed 3x with de-ionized water (to purify/isolate nanomaterial products and promote oxidation to cerium oxide). Particles were then re-suspended in fresh de-ionized water and ultra-sonicated for 20 min to disperse stable particles. Solutions were left standing overnight and any further sediment was removed by manually collecting supernatant of well-suspended particles. Particles were used without further modification.

#### 6.1.3 Characterization of CeONP^60/40^ and CeONP^20/80^ and analysis of SOD and CAT mimetic activity

Physicochemical characterization of CeONP^60/40^ and CeONP^20/80^ samples was performed using high resolution transmission electron microscopy (HRTEM; Philips Tecnai operating at 300 kV). X-ray photoelectron spectroscopy (XPS) was performed using a Thermo Scientific ESCALAB 250Xi spectrometer in an ultra-high vacuum (UHV) chamber (4 × 10^−9^ Torr). The radiation source was from a monochromatic Al-Kα cathode (binding energy: 1486 eV). The beam spot size was 650 μm and C1s peak at 284.6 eV was used as a base for binding energy calibration within an experimental error of ±0.2 eV. The quantitative determination of SOD-mimetic activity was performed using a SOD assay kit (Dojindo Molecular Technologies, kit #S311: SOD Assay Kit - WST). A xanthine/xanthine oxidase reaction system was used to estimate the superoxide anion scavenging activity. Similarly, the catalase-mimetic activity was determined using an Amplex red-based hydrogen peroxide assay kit (Invitrogen, Cat. No. # A22188, Carlsbad, CA). Each assay was performed per manufacturer instructions. All catalytic activity measurements were obtained using 2 mM CeO_2_ concentrations. Particle colloidal stability and related surface charging were determined *via* zeta potential measurements using a Zetasizer Nano (Malvern Instruments). Amine-reactive fluorescein 5(6)-isothiocyanate (FITC) labeling techniques and flow cytometry were used to confirm the cellular uptake of CeONPs at 24 h after CeONP treatment as previously described [69].

#### 6.1.4 Characterization of the speciation of CeO_2_ and CeO_2-x_ using density functional theory calculations

Calculations were performed using the VASP code [130,131], using a plane-wave basis set with cutoff 500eV and the projector augmented wave (PAW) approach [132,133]. The frozen core is [He] for O and [Xe] for Ce. The exchange correlation functional was the Perdew-Burke-Ernzerhof [134] (PBE) GGA. For the Ce f orbitals, we used the Liechtenstein methodology [135], where the coulombic (U) and exchange (J) parameters are treated as independent variables. We chose U = 5 eV and J = 0 eV, making the Liechtenstein and the Dudarev [136] methods equivalent. This methodology accounts for the presence of the localized Ce^3+^ states, and has been commonly used in the wider literature [55,64,65,137,138]. All calculations were spin polarized and 3D boundary conditions were used. The minimised bulk unit cell of CeO_2_ retain the space group 225 symmetry and has a lattice constant of 5.498 Å, which overestimates the experimental value of 5.411 Å [139] but is in line with previous literature [55,64,65,137,138]. The electronic and ionic convergence criteria were 1 × 10^−5^ eV and 1 × 10^−3^ eV Å^−1^. The Brillouin zone was sampled using a Γ-centred 5 × 5 x 5 k-point grid. Surfaces were generated using the METADISE code [140] and modelled using the slab method [141] in which two identical surfaces are created *via* the introduction of a vacuum gap perpendicular to the surface. A vacuum gap of 15 Å was used to minimize the interaction between images. The {100}, {110} and {111} slabs with a (2 × 2) expansion of the primitive surface unit cell were investigated. The {100} and {110} were 7 layers (28 CeO_2_ units) and the {111} were composed of 5 layers (20 CeO_2_ units). To remove the dipole of the {100} slab, half of the surface oxygen atoms were moved from one to the other side to the slab. The Brillouin zone sampled using a Γ-centred 2 × 2 × 1 grid, with the third vector perpendicular to the surface plane. Relaxation of the atomic structure for all atoms was deemed to have converged when the forces were below 1 × 10^−2^ eV Å^−1^. All slab calculations used symmetric introduction of oxygen vacancies and adsorbates on both sides of the slab, thus ensuring that the surfaces were identical, and the cell had no net dipole moment. As the number of configurations for adsorbed species on surfaces of ceria is extremely large, we have limited the choice to structures that maximize the hydrogen bonding between the adsorbate and the surface. The isolated H_2_O_2_ was simulated at the Γ point in a cubic cell of side 10 Å and with the same convergence criteria as the surface calculations. The energy of adsorption ($E_{Ads}$) of an adsorbate on ceria surfaces is calculated as $E_{Ads}=\frac{E_{Slab+Adsorbate}-\left(E_{Slab}+E_{Adsorbate}\right)}{2}$, where $E_{Slab+Adsorbate}$, $E_{Slab}$ and $E_{Adsorbate}$ are the energies of the adsorbate adsorbed onto the slab, of the bare slab, and of the adsorbate, respectively, and 2 accounts for the adsorption on adsorbates on both sides of the slab.

#### 6.1.5 Characterization of the isotropic and anisotropic activity and selectivity of CeONP^60/40^ and CeONP^20/80^ to O_2_^•–^

Isopropanol (IPA) and dimethyl sulfoxide (DMSO) were purchased from VWR. HPLC-grade water and potassium superoxide powder were purchased from Sigma-Aldrich. This spectrum was generated from dissolving KO_2_ in a 1:2:2 water/DMSO/IPA solution which forms a glass when cooled to 77 K. Each sample was initially at 40 mM KO_2_ but were allowed to sit for 45 s to allow the O_2_∸ to react with the CeONPs and/or solvent before being rapidly frozen in liquid N_2_, quenching any further reactions.

Following our previous work on superoxide [66] and cerium nanoparticles [142], solutions of H_2_O, DMSO and IPA in a 1:2:2 ratio, respectively, were prepared using aqueous CNPs with surface Ce^3+^/Ce^4+^ ratios both high (WB-CNPs) and low (TH-CNPs) and HPLC-grade H_2_O. The initial pH of the solutions was lowered to 3 to slow CNP precipitation (pH values rapidly rose upon KO_2_ dissolution to a final value of 9, leading to precipitation of the CNPs after approximately 1 min). A control was prepared similarly using only HPLC-grade H_2_O. KO_2_ powder stored in anhydrous conditions under N_2_ gas was then dissolved and the solution was allowed to sit for 45 s before being frozen in liquid nitrogen.

A Bruker EMXPplus-9.5/2.7/P/L X-band continuous wave EPR spectrometer was used for all EPR measurements and all O_2_∸ samples were run under identical conditions (100 kHz field modulation, 320 G scan range centred at 3280 G, 3.2 G modulation amplitude and microwave power of 45 dB at 77K).

## 7 *In vitro* studies using hBMSCs and murine RAW 264.7 macrophages following exposure to ionizing radiation

### 7.1 Culture and irradiation of cells

Human bone marrow-derived mesenchymal stromal cells (ATCC® PCS-500-012™) were maintained in Dulbecco's Modified Eagle's Medium (DMEM; Thermo Fisher Scientific, USA) containing 10% fetal bovine serum (FBS; Thermo Fisher Scientific, USA), and 1% penicillin/streptomycin (Thermo Fisher Scientific, USA) in a humidified incubator with 5% CO_2_ at 37 °C. Murine-derived macrophages (RAW 264.7 cells, ATCC® TIB-71™) were maintained in DMEM supplemented with 10% heat inactivated FBS, and 1% (v/v) penicillin/streptomycin in a humidified incubator containing 5% CO_2_ at 37 °C. To investigate the protective effect of CeONP^60/40^ and CeONP^20/80^ on IR-induced cellular damage, cells were pre-treated at a dose of either 1 μg/mL or 10 μg/mL of CeONPs 24 h prior to IR exposure. The dosage of CeONP^60/40^ or CeONP^20/80^ were selected based on our previous study [59]. After removal of the CeONPs through washing with phosphate buffered saline (PBS) thrice, cells were subjected to irradiation. Cells were exposed to an IR (X-ray) dose of 7 Gy, a 160 kV tube voltage, 4 mA tube current, at a distance of 30 cm between the source and the surface (SC 500 smart controller, KIMTRON, USA). After X-ray or mock-X-ray exposure, the media containing either the hBMSCs or RAW264.7 cells, were replenished with 1 μg/mL or 10 μg/mL of CeONP^60/40^ or CeONP^20/80^. Cells in the control group received no CeONP treatment.

### 7.2 Analyses of intracellular ROS generation in hBMSCs, and the radical scavenging properties of CeONP^60/40^ and CeONP^20/80^

Intercellular ROS formation was assessed using a DCFDA/H2DCFDA- Cellular ROS Assay Kit (ab113851, Abcam, USA) and performed according to the manufacturer's instructions. Cells were counter-stained with MitoSpy™ Red CMXRos (Biolegend, USA). Qualitative assessment of CeONP^60/40^ or CeONP^20/80^ scavenging was assessed on day 1 following radiation, and using confocal laser scanning microscope imaging (Zeiss, USA). Intracellular superoxide anions (O_2_^·−^) was measured using a MitoSOX™ Red Mitochondrial Superoxide Indicator kit (M36008, Thermo Fisher Scientific, USA). Superoxide anion scavenging was quantified on day 1 using a CytoFLEX Flow Cytometer (Beckman Coulter, USA). SOD1/Cu–Zn SOD Antibody (NBP2-24915, Novus Biological, USA) and α-Tubulin (NB100–690SS, Novus Biologicals, USA) were used for quantifying SOD1 expression. Catalase activity was determined using an Amplite® Fluorimetric Catalase Assay Kit (11306, AAT Bioquest, USA) and performed per manufacturer instructions.

### 7.3 Quantitative real-time reverse-transcription polymerase chain reaction (qRT-PCR) in hBMSCs and murine RAW 264.7 macrophages

Here we used qRT-PCR to investigate the gene expression of endogenous antioxidant factors in hBMSCs, and the pro-inflammatory and pro-osteoclastic gene expression in murine macrophages following IR-induced damage. We determined the protective effect of CeONP^60/40^ or CeONP^20/80^ to these cells. Total RNA was extracted using a PureLink™ RNA Mini Kit (12183018A, Thermo Fisher Scientific, USA) and genomic DNA contamination removed from samples using DNase (PureLink™ DNase Set, 12185010, Thermo Fisher Scientific, USA). RNA concentration was measured using a NanoDrop 8000 spectrophotometer (NanoDrop technologies). 500 ng of total RNA was used as a template for reverse transcription and cDNA synthesis was performed using SuperScript™ III First-Strand Synthesis SuperMix (18080400, Thermo Fisher Scientific, USA). qRT-PCR was performed using Fast SYBR™ Green Master Mix (4385612, Thermo Fisher Scientific, USA) on an ABI Prism 7500 Thermal Cycler (Applied Biosystems, Foster City, California, USA). The primers used in this study were KiCqStart™ Primers and were purchased from Millipore Sigma. The fold change of relative mRNA expression was calculated using the comparative Ct (2^−ΔΔCT^) method. We used these predesigned qRT-PCR assays for the analysis of *Catalase (Gene ID: 847), GPX4 (Gene ID: 2879), SOD1(Gene ID: 6647), and SOD2 (Gene ID: 6648)* in hBMSCs, and *IL-1β (Gene ID: 16176), IL-6 (Gene ID: 16193), RANKL (Gene ID: 21943)* and *CTSK (Gene ID: 13038)* as determinants of the osteoclastic differentiation of macrophages.

### 7.4 Analyses of CeONP^60/40^ and CeONP^20/80^ protection against IR-induced DNA damage in hBMSCs

To investigate cellular DNA damage following exposure to radiation and the effect of the nanozyme formulations, a comet assay® (4250-050-K, R&D Systems, USA) was performed as previously described [69], and on day 3 after irradiation. In brief, the cells were detached gently using a cell scraper, pelleted and then resuspended in ice cold PBS, free of Ca^++^ and Mg^++^ ions. A 50 μL cell suspension was mixed with 500 μL molten LM Agarose at 37 °C. 50 μl of the mixture was then pipetted immediately onto a CometSlide™. After a 10 min incubation period in the dark and at 4 °C, the slides were immersed in a lysis buffer overnight at 4 °C. Cells were then immersed for 20 min in a freshly prepared alkaline unwinding solution in the dark and at room temperature before performing gel electrophoresis at 21 V for 30 min. The slides were then washed twice in deionized H_2_O, followed by rinsing once in 70% ethanol. Samples were stained using 100 μL of diluted SYBR™ Green I Nucleic Acid Gel Stain for 10 min in the dark. After rinsing briefly in water, slides were dried at 37 °C and images were taken using a confocal laser scanning microscopy (Zeiss, USA).

### 7.5 Analyses of CeONP^60/40^ and CeONP^20/80^ protection against IR-induced cellular senescence in hBMSCs

Cellular senescence in hBMSCs was performed using a senescence β-galactosidase (β-gal) staining kit (9860, Cell Signaling Technology, USA). Briefly, hBMSCs were fixed at room temperature 28 days post-irradiation. After washing, cells were incubated with β-galactosidase staining solution overnight at 37 °C. Images were captured using an inverted phase microscope (BZ-X800E, Keyence, USA).

### 7.6 Analyses of CeONP^60/40^ and CeONP^20/80^ on cell proliferation and actin filament and nuclear morphology

An MTT [3-(4, 5-dimethylthiazol-2-yl)-2, 5-diphenyl tetrazolium bromide] assay (M2128, Millipore Sigma, USA) was performed and used according to the manufacturer's instructions. In brief, 20 μL of a 5 mg/mL MTT solution in PBS was added into each well for 4 h. The supernatant was then carefully removed and 100 μL of dimethyl sulfoxide (DMSO; Thermo Fisher Scientific, USA) was used to dissolve the formazan crystals. Absorbance was measured using a microplate reader (Synergy HTX, USA) at 570 nm. To evaluate changes in cell morphology, cells were fixed with 4% paraformaldehyde for 20 min. The cytoskeleton and nuclei were stained using Alexa Fluor™ 488 Phalloidin (A12379, Thermo Fisher Scientific, USA) and 4′,6-Diamidino-2-phenylindole dihydrochloride (DAPI; D9542, Millipore Sigma, USA), respectively. Images were captured using confocal laser scanning microscopy (Zeiss, USA).

### 7.7 Analyses of CeONP^60/40^ and CeONP^20/80^ on osteoclastic formation using murine RAW 264.7 macrophages

Tartrate resistant alkaline phosphatase (TRAP) staining was used to determine the formation of osteoclasts from macrophages. Following radiation-induced damage, RAW 264.7 macrophages were cultured in DMEM containing 10% FBS and supplemented with or without CeONPs in a humidified incubator containing 5% CO_2_ at 37 °C for 3 days. Cells were fixed with 4% paraformaldehyde for 20 min. Images were captured using an inverted phase microscope (BZ-X800E, Keyence, USA).

### 7.8 Analyses of CeONP^60/40^ and CeONP^20/80^ on the osteogenic differentiation of hBMSCs and mineralization following IR-induced damage

After CeONP^60/40^ or CeONP^20/80^ pre-treatment and following exposure to 7Gy of radiation, the response of the hBMSCs to osteogenic induction was performed using DMEM containing 10% FBS supplemented with the osteogenic components; 2 mM β-glycerophosphate, 100 μM l-ascorbic acid 2-phosphate, 10 nM dexamethasone (Sigma-Aldrich, USA). The CeONPs were introduced at a dose of either 1 μg/mL or 10 μg/mL and the media was changed every 3 days. To determine the cell response during osteogenic differentiation, alkaline phosphatase (ALP) activity was measured using ALP staining and Western blot, gene expression of osteogenic-related markers was evaluated by qRT-PCR, and mineral deposition was quantified using alizarin S red staining. ALP staining was performed at 14 days and according to the manufacturer's instructions (K2035-50, BioVision, USA). In brief, after removing the culture medium, cells were washed in 300 μL washing buffer, and stained with ALP staining solution for 30 min at 37 °C. After rinsing twice with washing buffer, images were captured using an inverted phase microscope (BZ-X800E, Keyence, USA). Gene expression of osteogenic-related markers was evaluated by qRT-PCR based on our previous study [69]. ALP (NBP2-67295, Novus Biological, USA) and α-Tubulin (NB100–690SS, Novus Biologicals, USA) were used for quantifying ALP expression. ALP Western blot was performed based on our previous study [69]. Alizarin red S staining (A5533-25G, Millipore Sigma, USA) was performed to quantify the deposition of mineral noduli after 28 days of culture. In brief, cells were fixed using 4% paraformaldehyde for 20 min at room temperature. After fixation, cells were washed with deionized H_2_O and incubated with 2% pH 4.1 alizarin red S solution for 20 min at room temperature. The samples were air-dried at room temperature. Images were captured using an inverted phase microscope (BZ-X800E, Keyence, USA). To quantify mineralization, the noduli were dissolved using 10% cetylpyridinium chloride and analyzed by determining Optical Density (OD) values at 562 nm.

### 7.9 Analysis of CeONP^60/40^ and CeONP^20/80^ on HIF1α expression in hBMSCs

To evaluate the levels of and HIF-1α expression, a HIF1α human ELISA kit (EHIF1A, Thermo Fisher Scientific, USA) was used according to the manufacturer's instructions. In brief, hBMSCs were pre-treated with 10 μg/mL of CeONPs for 24 h. After the removal of CeONPs through washing with PBS thrice, cells were subjected to irradiation. After X-ray exposure, hBMSCs were replenished with 10 μg/mL of CeONPs in osteogenic induction medium and cultured in a hypoxia incubator (5% oxygen) for 2 h. The absorbance was read at 450 nm using a microplate absorbance spectrophotometer (Synergy HTX, USA). The concentrations of HIF1α were calculated using the standard calibration curve.

## 8 *In vivo* studies and analysis of CeONP^60/40^ in 9-week-old SAS Sprague-Dawley rats and following IR-induced tissue damage

### 8.1 Power calculation and randomization of rat allocation to experimental group

The predesigned primary endpoint in the rat studies was to record the effect of CeONP^60/40^ in protecting against trabecular and cortical bone tissue loss following exposure to radiation-induced damage. Our power analysis suggested that when using six rats in each experimental group, we would have 80% power to detect a biologically significant effect in bone loss, and therefore we used six animals in this study. Animals were randomly assigned to each experimental group and assessments of the outcomes from these experiments were blinded to the investigators.

### 8.2 In vivo study design, CeONP^60/40^ administration, and exposure of the rat hind limb to ionizing radiation

Thirty-six male SAS Sprague Dawley rats aged 8–9 weeks and weighing ∼200g, were used in the *in vivo* study. Animals were acclimatized for a period of 1 week before commencement of the experiment. The handling of the animals was approved by the Institutional Animal Care and Use Committee of University of Central Florida (2020–48). All animal treatments were performed as per the CITI protocols for the University of Central Florida and NIH guidelines. Rats were randomly divided into three experimental groups: (1) mock-X-ray control group, (2) X-ray only group and, (3) X-ray + CeONP^60/40^ given group. Rats were euthanized at either 7- or 14-days following the first of three doses of radiation. To prepare the animals for exposure to radiation, general anaesthesia was induced and maintained with 2% isoflurane and an oxygen flow rate of 0.3 mL/min. The main body of the rats were shielded by a radiation protection lead blanket (MPS-S, Z&Z Medical, USA), and the hind limbs of the rat were subjected to local fractionated irradiation (8 Gy/each time) on days 1, 3 and 5 of the study (a total of 24 Gy was applied), at a 160 kV tube voltage, a 4 mA tube current, and at a distance of 30 cm between the source and the surface of the animal (SC 500 smart controller, KIMTRON, USA). Rats received CeONP^60/40^ at a dose of 4 mg/kg while suspended in sterile saline and 24 h prior to the first dose of radiation (day zero). A second dose of CeONP^60/40^ was given on day 4. The CeONPs^60/40^ were administered intravenously and *via* tail vein injection.

### 8.3 Analyses of CeONP^60/40^ organ toxicity

In the animals euthanized on day 14, sections of the liver, pancreas and kidney were obtained and prepared for histological analysis using standard techniques [143]. Hematoxylin and eosin staining was performed according to the manufacturer's instructions (ab245880, Abcam, USA).

### 8.4 Analyses of CeONP^60/40^ and *in vivo* DNA damage to cells within the bone marrow niche

On day 7 after irradiation, DNA damage to bone marrow cells was detected using the comet assay and as described above.

### 8.5 Whole-mount histological staining

Dissection of the femora was undertaken for detection of bony elements by hematoxylin and eosin (H&E) staining. Standard histology methods were used [143]. Briefly, samples were fixed, dehydrated in graded ethanol, and embedded in paraffin wax. Five μm thick sections were prepared from paraffin-embedded samples, followed by deparaffinising and rehydrating. Then, the tissue sections were stained with H&E solution. Longitudinal sections were prepared through the distal femoral condyle. Images were captured using an inverted phase microscope (BZ-X800E, Keyence, USA). To quantify bone area (%), trabecular bone area (BA) to total tissue area (TA) was calculated from 5 random slides in each group at the same magnification (×10) using Imagej software.

### 8.6 Analyses of CeONP^60/40^ on the cellular expression of RANKL

Immunohistochemical staining was performed according to the Abcam protocol (https://www.abcam.com/ps/pdf/protocols/ihc_p.pdf). Briefly, sections were deparaffinised in xylene, hydrated in descending alcohol. Slides were boiled in 10 mM sodium citrate buffer (pH 6.0) for 10 min to retrieve antigenicity and then cooled down at room temperature for 20 min. The endogenous peroxidase activity was inactivated by a hydrogen peroxide blocking reagent (ab64218, Abcam, USA) for 10 min. After washing in PBS, sections were treated with a protein block (ab64226, Abcam, USA) at room temperature for 30 min. Immunostaining was performed overnight with the following primary antibodies diluted in antibody diluent (ab64211, Abcam, USA) at 4 °C overnight: RANKL (NB100-56512, Novus Biologicals, USA). Goat anti-Mouse IgG Secondary Antibody [HRP Polymer] (VC001-025, Novus Biologicals, USA) was used as secondary antibody. The slides were stained with SignalStain® DAB Substrate Kit (#8059; Cell Signaling Technology, USA) and counterstained with hematoxylin. All stained sections were dehydrated in graded alcohol baths of increasing concentration, cleared in xylene, and coverslipped with VectaMount™ AQ mounting medium (NC9354983, Fisher Scientific, USA). Images were captured using an inverted phase microscope (BZ-X800E, Keyence, USA).

### 8.7 Analyses of CeONP^60/40^ on osteoclastic activity

Tartrate resistant alkaline phosphatase staining was performed to determine osteoclastic activity using the standard naphthol AS-BI phosphate post-coupling method. Briefly, tissue sections were rehydrated and incubated with TRAP staining solution containing 0.2 M sodium acetate buffer (pH 5.0), 50 mM L-(+) Tartaric acid, 0.5 mg/mL naphthol AS-MX phosphate, and 1.1 mg/mL Fast Red TR Salt 1,5-naphthalenedisulfonate salt (ab146351, Abcam, USA) for 1–3 h at 37 °C. Nuclei were then counterstained with hematoxylin for 5 min before mounting with VectaMount™ AQ mounting medium (NC9354983, Fisher Scientific, USA). Images were captured using an inverted phase microscope (BZ-X800E, Keyence, USA). To further investigate the effect of CeONP^60/40^ on bone resorption, bone resorption marker C-terminal end of the telopeptide of type I collagen (CTX-1) was measured in rat sera by ELISA (NBP2-69077, Novus Biologicals, USA) per manufacturer instructions.

### 8.8 Analyses of CeONP^60/40^ on cellular senescence

Frozen tissue sections (20 μm) from decalcified samples were used for detection of β-gal activity using the methods as described above. Nuclear fast red (50-317-51, Electron Microscopy Sciences, USA) was used for counterstaining.

### 8.9 Nano-CT scanning

Tibia was prepared for high-resolution -ray computed tomography (CT-scanning) using GE V∣TOME∣X M 240 Nano CT scanner (General Electric) at the University of Florida (Gainesville, FL, USA) with a 180 kv x-ray tube with a diamond-tungsten target and with the following settings: 75 kV, 150 mA, a 0.5 s detector time, averaging of three images per rotation and a voxel resolution of 12.4 μm 3D models of the trabecular network within the proximal tibia were created using a 3D Slicer (v4.11.20210226; Brigham and Women's Hospital and Massachusetts Institute of Technology). The DICOM files were imported, and a label map created. A threshold was used to automate the segmentation process and a smooth crushing tool used to manually clean the segments.

## 9 Analyses of CeONP^60/40^ and changes in bone strength

### 9.1 Three-point bending analyses of SAS rats

Immediately after the dissection, tibiae retrieved on day-7 were stored at −20 °C. Three-point-bending tests were performed using a universal testing machine within 1 month of freezing (Criterion® 43, MTS, Minnesota, USA). Each tibia was loaded to failure at a displacement rate of 0.02 mm/s. The distance between the support bars of the 3-point-bending fixture was 8 mm and each tibia was positioned horizontally with the anterior bow lying superiorly (Supplementary Fig. S10). A vertical force was applied to the tibial mid-shaft using a 3 mm diameter loading roller until failure occurred. Load-displacement curves were obtained, and the mechanical properties were calculated as follows:

(1) $$\sigma =\frac{FLc_{o}}{4I}$$

where σ is the stress (Pa), F is the applied load (N), L = 0.008 is the span distance between the supports (m), $c_{o}$ is the outer radius of the tibia's midshaft (m), and I is the moment of inertia (m^4^) calculated as follows:

(2) $$I=\frac{\pi }{4\left(c_{o}^{4}-c_{i}^{4}\right)}$$

where $c_{i}$ is the inner radius of the tibia's midshaft (m). The morphological parameters of three bones from each group were obtained following computed tomography (CT). Micro-computed tomography (μCT) scans were performed using a cone beam scanner (GE Phoenix Nanotom-M™, Waygate Technologies). Formalin fixed tibiae retrieved on day-7 were placed in 15 mL Eppendorf tubes and imaged at a 90 kV source voltage, 110 μA source current (mode 0) using a tungsten-diamond target with a 500 ms exposure time at 7–9 μm isotropic voxel resolution (depending on femur size). Data was collected for 1080 projections over 360° (0.33° steps) with three averaged images per rotation position. Four cross-sections were selected from the same region of the proximal, mid and distal tibia, and both inner and outer diameters were calculated from each cross-section. The average value of inner-to-outer diameter from each cross-section were calculated to determine the inner radius. Ultimate stress, and fracture stress were subsequently quantified.

### 9.2 Statistical analyses

All numerical data is presented as mean ± standard deviation (SD). Statistical analysis was carried out using GraphPad Prism (version 8.0, US) and groups were compared using one-way analysis of variance (ANOVA) and a post-hoc Mann Whitney *U* test. *p* values < 0.05 were considered significant.

## CRediT author statement

All authors made substantial contributions to conception and design, acquisition of data, or analysis and interpretation of data.

## Declaration of competing interest

The authors declare that they have no known competing financial interests or personal relationships that could have appeared to influence the work reported in this paper.
