# Supplementary material for: A novel approach for the prevention of ionizing radiation-induced bone loss using a designer multifunctional cerium oxide nanozyme
Source: Bioact Mater. 2022 Sep 21;21:547–65. doi: 10.1016/j.bioactmat.2022.09.011 (PMC9507991; doi:10.1016/j.bioactmat.2022.09.011)
Supplement: Multimedia component 1 [file mmc1.docx]

**Supplementary Information**

**A novel approach for the prevention of ionizing radiation-induced bone loss using a designer multifunctional cerium oxide nanozyme**

Fei Wei, Craig J. Neal, Tamil Selvan Sakthivel, Yifei Fu, Mahmoud Omer, Amitava Adhikary, Samuel Ward, Khoa Minh Ta, Samuel Moxon, Marco Molinari, Sergey N. Yarmolenko, Vee San Cheong, Nina Orlovskaya, Ranajay Ghosh_,_ Sudipta Seal* and Melanie J. Coathup*

*Seal and Coathup are co-senior authors

**Supplementary Figure S1**

**
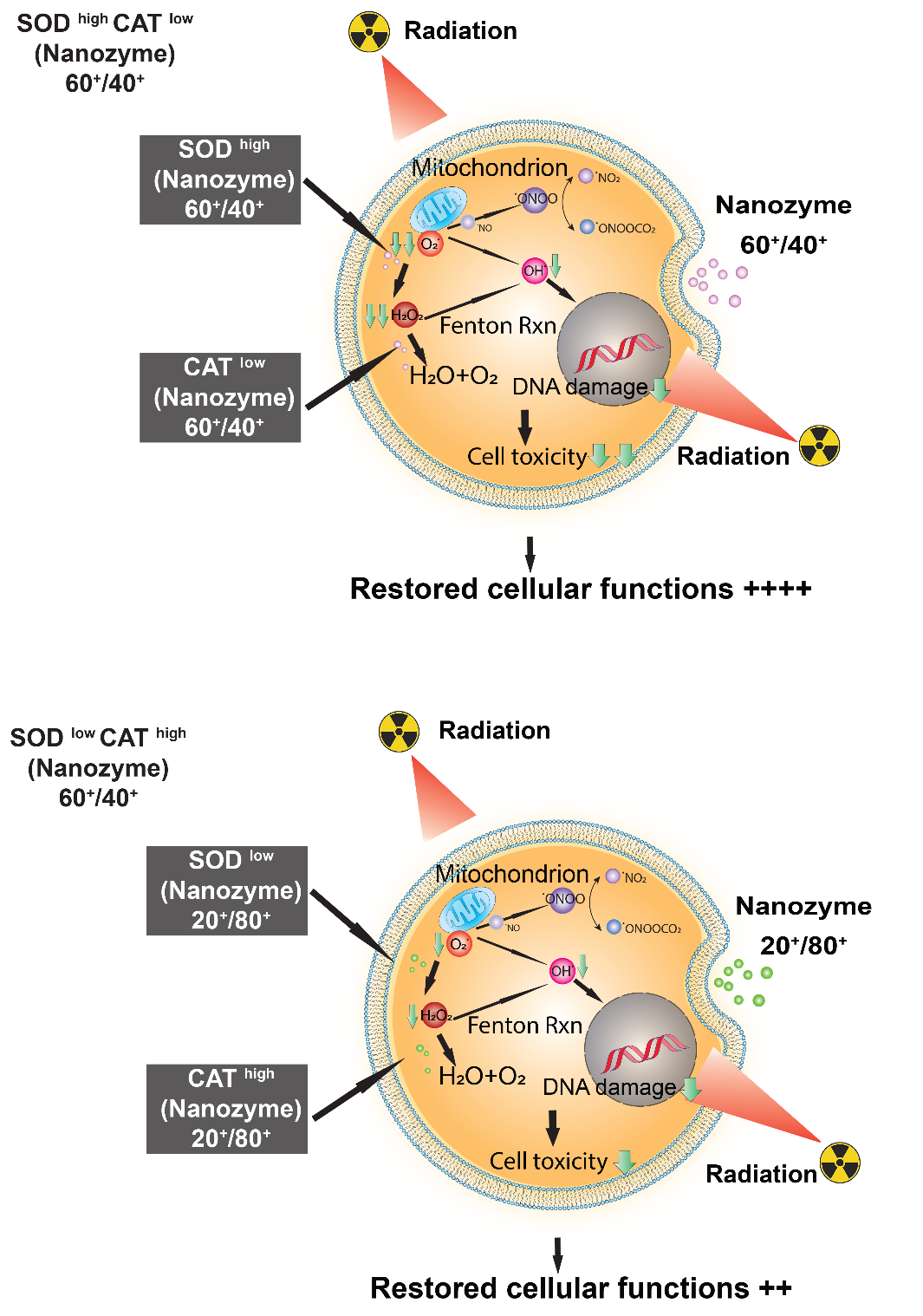
**

**Supplementary Figure S1. CeONP regulation of ROS following exposure to IR.** The O_2_^•-^ anion is the most common ROS and a precursor to many other harmful ROS. H_2_O_2_ is a precursor to OH^•^ *via* the Fenton reaction. Through its well-established CAT- and SOD-mimetic activities, CeONPs are able to scavenge ROS and therefore afford the potential of reducing the generation of O_2_^•-^, H_2_O_2_ and OH^•^ radicals. *In vitro* studies have shown human bone marrow-derived mesenchymal stem cells (hBMSCs) to exhibit a dose-dependent decrease in proliferation and a reduced capacity for osteogenic differentiation following exposure to IR (39–41). Osteoblasts display growth inhibition and reduced bone mineral deposition (42,43) and macrophages when either directly (44) or indirectly damaged by IR, exert their bystander effect by releasing high concentrations of proinflammatory cytokines that serve to further suppress osteoblastic activity and stimulate osteoclastogenesis and bone resorption (45–47). The combined dysfunction caused by IR to BMSCs, osteoblasts, immune cells and endothelial cells (48), represents the primary contributor for increased bone loss following exposure to IR (49). Downregulating the direct and indirect damage to cells during and immediately following IR exposure, may limit the subsequent dysfunction and tissue damage observed.

**
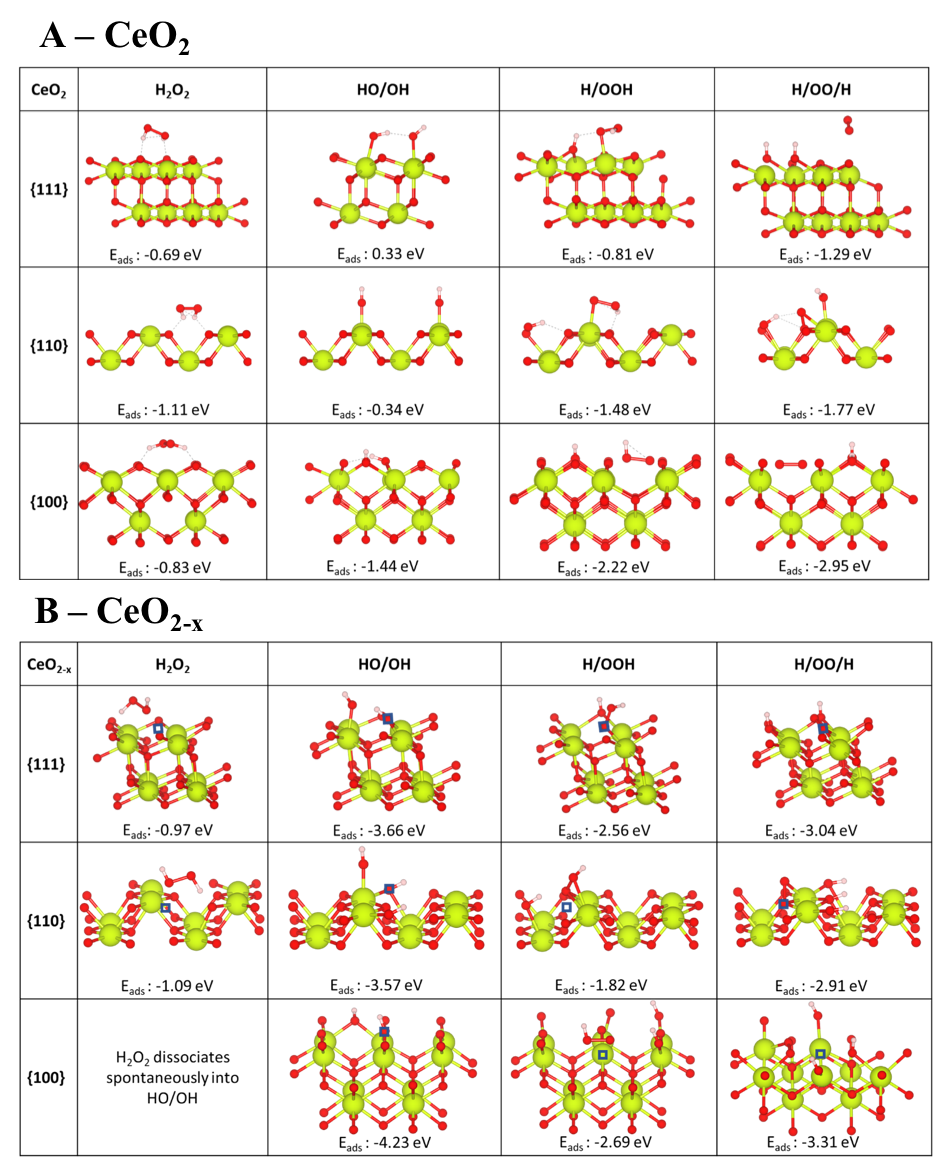
Supplementary Figure S2**

**Supplementary Figure S2.** Adsorption energies of molecular H_2_O_2_ and its dissociation products (HO/OH, H/OOH, H/OO/H) onto the {111}, {110} and {100} surfaces of stoichiometric CeO_2_ and reduced CeO_2-x_ calculated using DFT simulations. Ce, O and H as yellow, red, and white spheres, and oxygen vacancies as blue squares.

**Supplementary Figure S3**

**
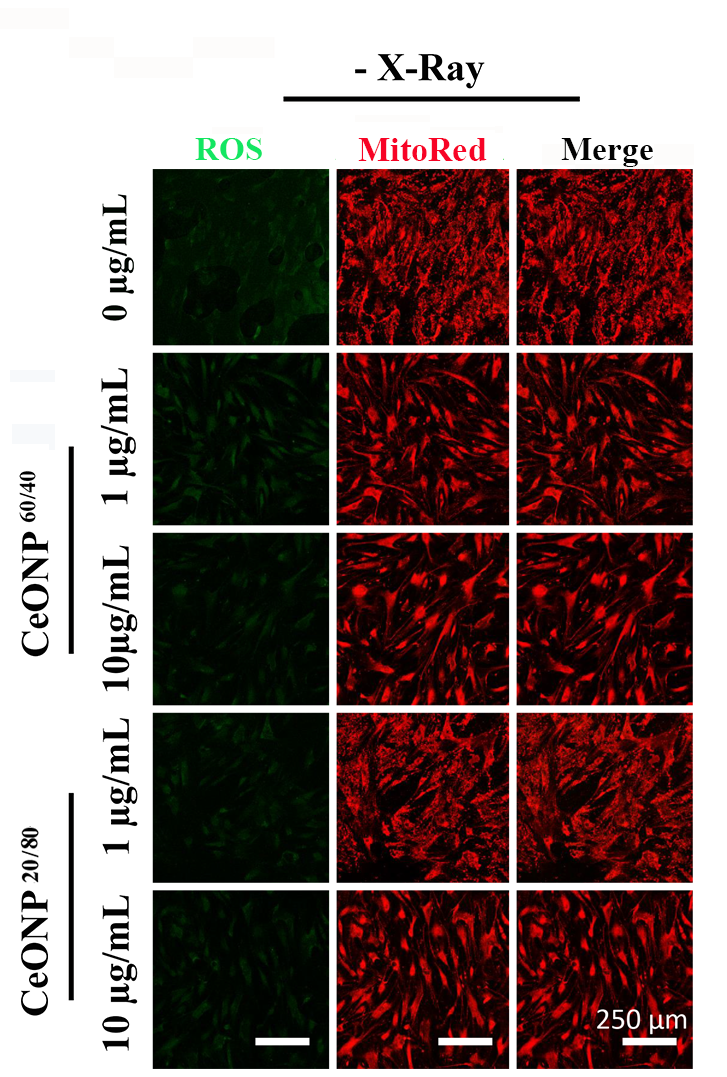
**

**Supplementary Figure S3. CeONP^60/40^ and CeONP^20/80^ pre-treatment to primary hBMSCs in the absence of irradiation treatment had no effect on intracellular ROS generation.** Representative confocal micrographs of intracellular ROS staining in each of the experimental groups are presented. After 24h pre-treatment with CeONPs at a dose of 1 and 10 μg/mL followed by mock exposure to irradiation, cells were stained with ROS/DCFDA (green), and counter-stained with MitoSpy™ Red CMXRos (red) indicating the presence of mitochondria in living cells. Cells were assessed 24h later. ROS production was limited in all groups demonstrating limited ROS generation by hBMSCs cells following treatment with both doses and formulations of CeONPs and when in the absence of radiation. Experiments were carried out in triplicate.

**Supplementary Figure S4**


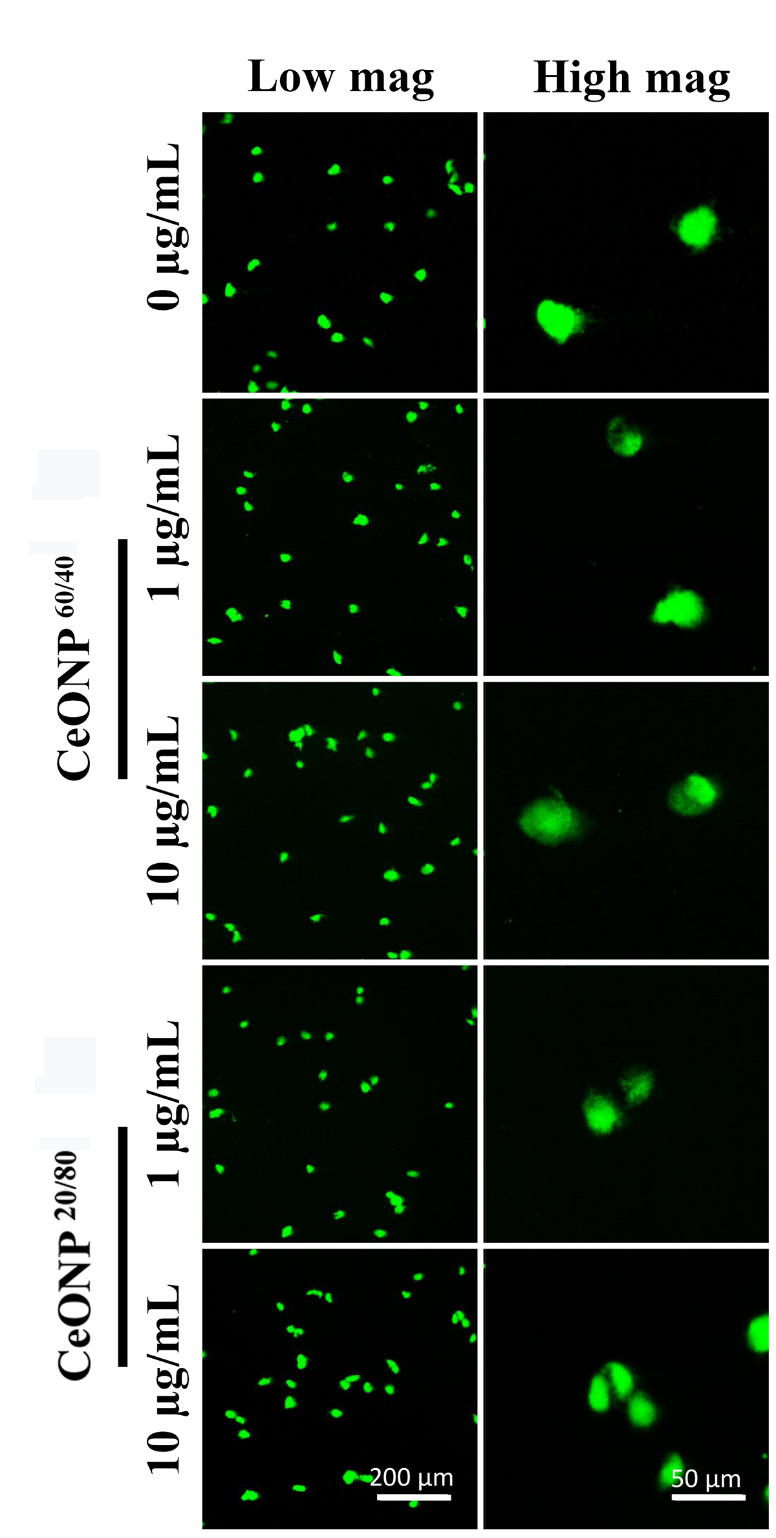


**Supplementary Figure S4. CeONP^60/40^ and CeONP^20/80^ pre-treatment to primary hBMSCs in the absence of irradiation displayed no evidence of DNA damage.** Representative confocal micrographs of DNA damage (Comet Assay®) 3 days after mock exposure are presented. hBMSCs were pre-treated with each nanozyme at a dose of 1 and 10 μg/mL, for 24 h and prior to mock IR exposure. Images were captured using confocal laser scanning microscopy. The characteristic “comet tail” that represents DNA fragmentation was not observed, indicating limited DNA damage when cells were treated with both doses and formulations CeONPs and when in the absence of radiation. Experiments were carried out in triplicate.


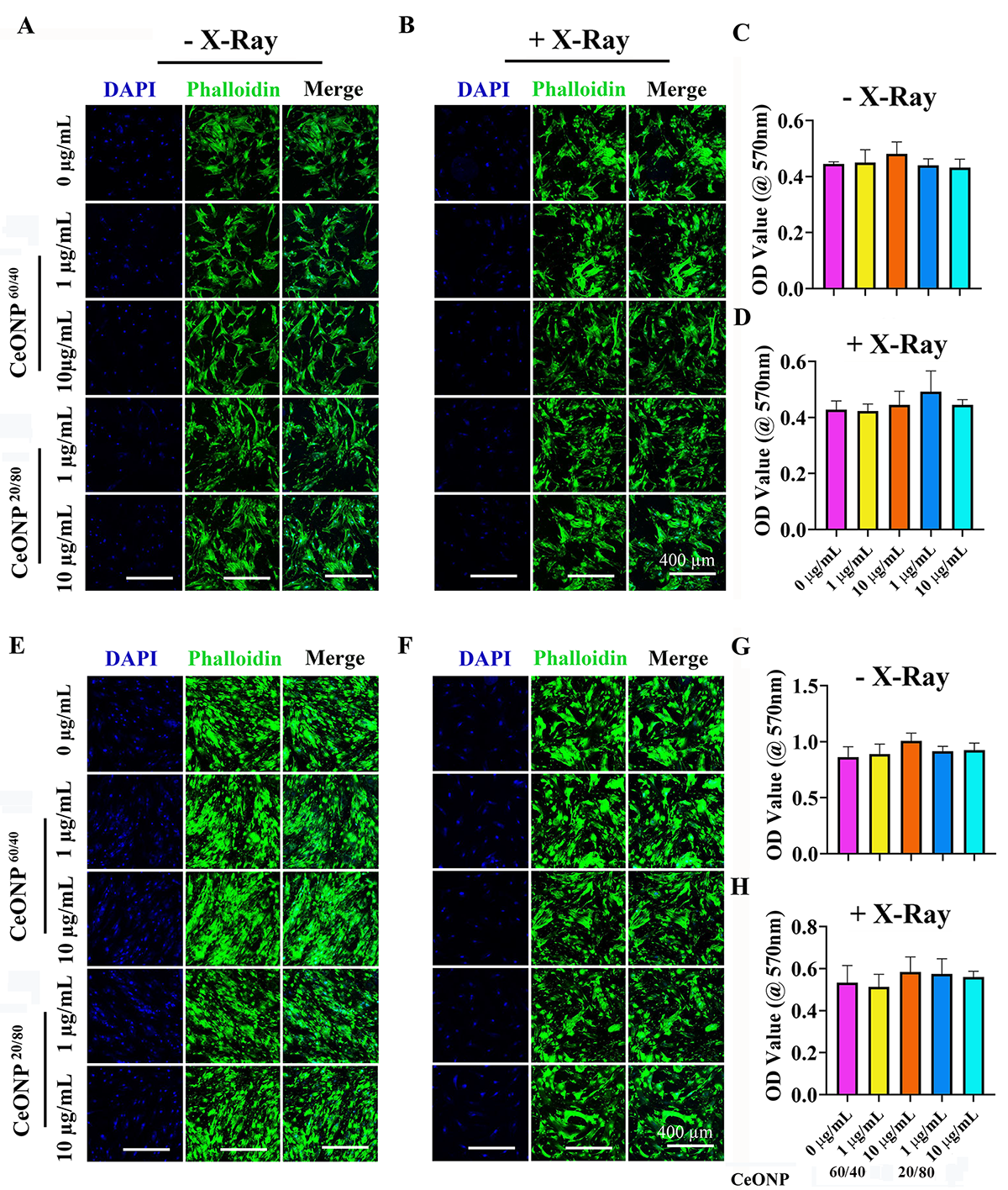
**Supplementary Figure S5**

**Supplementary Figure S5. Exposure of primary hMSCs to 7Gy of irradiation (X-ray) decreases cell proliferation on day 5 but not day 3, following exposure. Pre-treatment of cells with 1 or 10 μg/mL of CeONP^60/40^ and CeONP^20/80^ showed no effect on the rate of proliferation and cytoskeletal morphology over this time period.** [A-B] Representative confocal micrographs of hBMSCs following mock-X-ray exposure (A) and X-ray exposure (B), 3-days post-irradiation and following 24h pre-treatment with 1 or 10 μg/mL of CeONPs. Cells were fixed and the actin filaments and nuclei were stained with phalloidin (green) and DAPI (blue) respectively, prior to examination using confocal laser scanning microscopy 3-days post-irradiation. Qualitative analysis of cells following treatment with both formulations showed no apparent changes in nuclear morphology or actin filament architecture when compared with cells in the untreated, unexposed cells. [C-D] Cell proliferation was quantified using an MTT assay and following mock-IR exposure (C) and X-ray exposure (D) group showed no significant differences in absorbance values. [E-F] Representative confocal micrographs of hBMSCs at 5 days post mock-X-ray exposure (E) and X-ray exposure (F). [G-H] A graph demonstrating cell proliferation following mock-exposure (G) and X-ray exposure (H). The CeONPs were replenished in the media in all relevant groups. Experiments were carried out in triplicate. All values are given as the mean ± SD.

**Supplementary Figure S6**

**
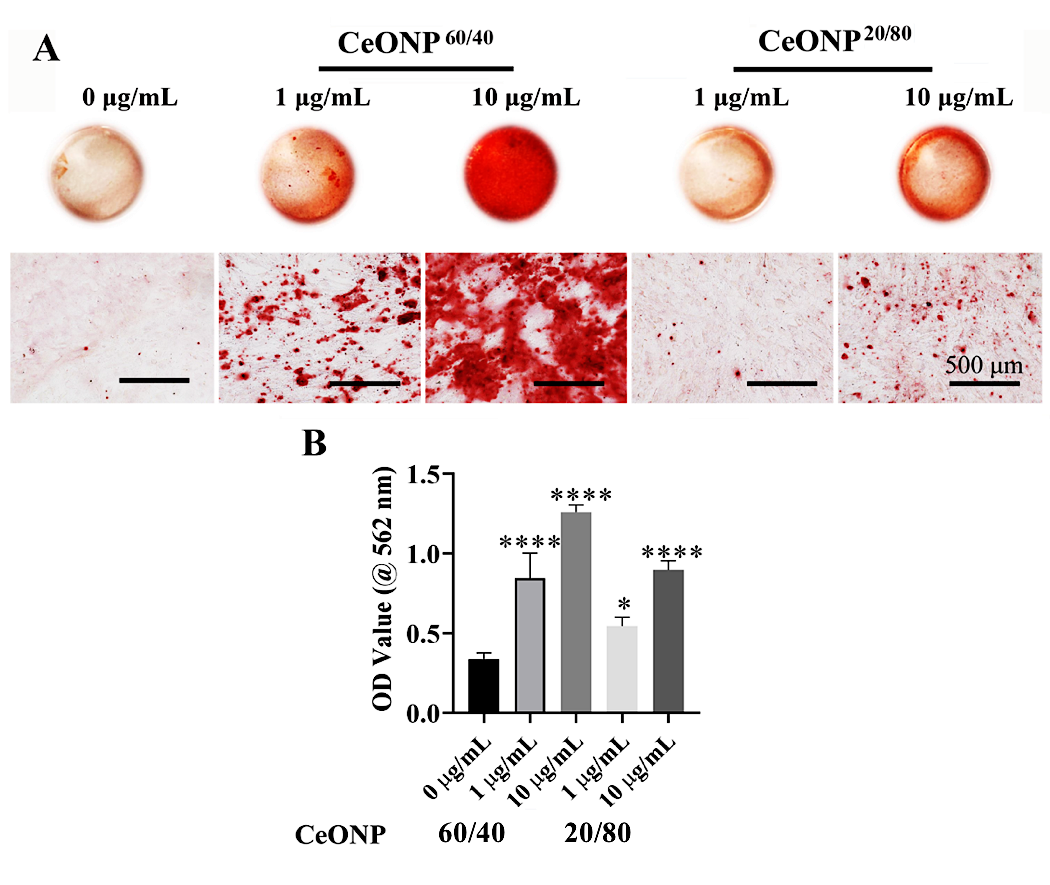
**

**Supplementary Figure S6. CeONP^60/40^ and CeONP^20/80^ pre-treatment to primary hBMSCs in the absence of irradiation increased hBMSC mineralization**. [A] hBMSCs pre-treated with 1 and 10 μg/mL of CeONP^60/40^ or CeONP^20/80^ followed by mock exposure. Cells were cultured in osteogenic media over a period of 28-days. Alizarin Red Staining was performed to determine mineral deposition. Areas of red indicate regions of mineralization. [B] Mineral deposition in the absence of radiation was quantified. Both CeONP^60/40^ and CeONP^20/80^ at a dose of 1 and 10 μg/mL, significantly increased the differentiation of primary hBMSCs and mineral deposition. This effect was further promoted in cells treated with 1 and 10 μg/mL of the nanozyme, CeONP^60/40^. The CeONPs were replenished in the media and experiments were carried out in triplicate. All values are given as the mean ± SD. Statistics presented compare each experimental group with the control (0 μg/mL) group of cells. **p* < 0.05, *****p* < 0.0001.


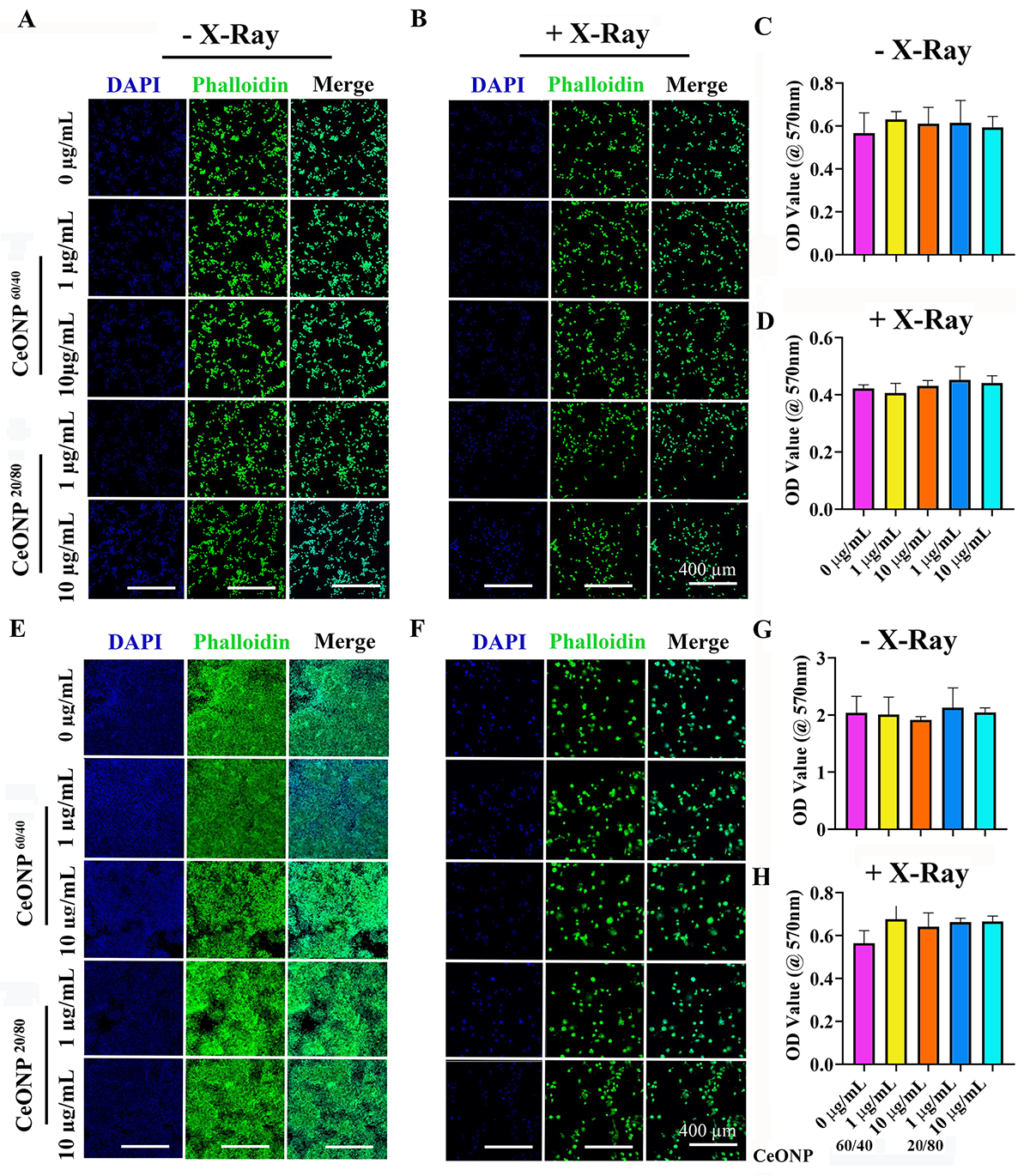
**Supplementary Figure S7**

**Supplementary Figure S7. Exposure of RAW 264.7 macrophages to 7 Gy of irradiation (X-ray) decreases cell proliferation on both day 1 and 3 following exposure. Pre-treatment of cells with 1 or 10 μg/mL of CeONP^60/40^ and CeONP^20/80^ demonstrated no effect on the rate of proliferation and cytoskeletal morphology.** [A-B] Representative confocal micrographs of RAW264.7 pre-treated with either 0, 1 or 10 μg/mL of CeONPs following mock-X-ray exposure (A) or X-ray exposure (B). Images were taken at 1-day post-irradiation. [B] A graph demonstrating cell proliferation following mock-X-ray exposure (C) and X-ray exposure (D). [E-F] Representative confocal micrographs of RAW264.7 at 3 days following mock-X-ray exposure (E) and X-ray exposure (F). [G-H] Cell proliferation was determined in mock-X-ray exposure (G) and X-ray exposure (H) RAW264.7 cells. Qualitative analysis of cells following treatment with both formulations showed no apparent changes in nuclear morphology or actin filament architecture on days 1 and 3 and when compared with cells in the untreated, unexposed cells. Using an MTT assay, proliferation was quantified and no significant differences were found. Experiments were carried out in triplicate. All values are given as the mean ± SD.

**Supplementary Figure S8**


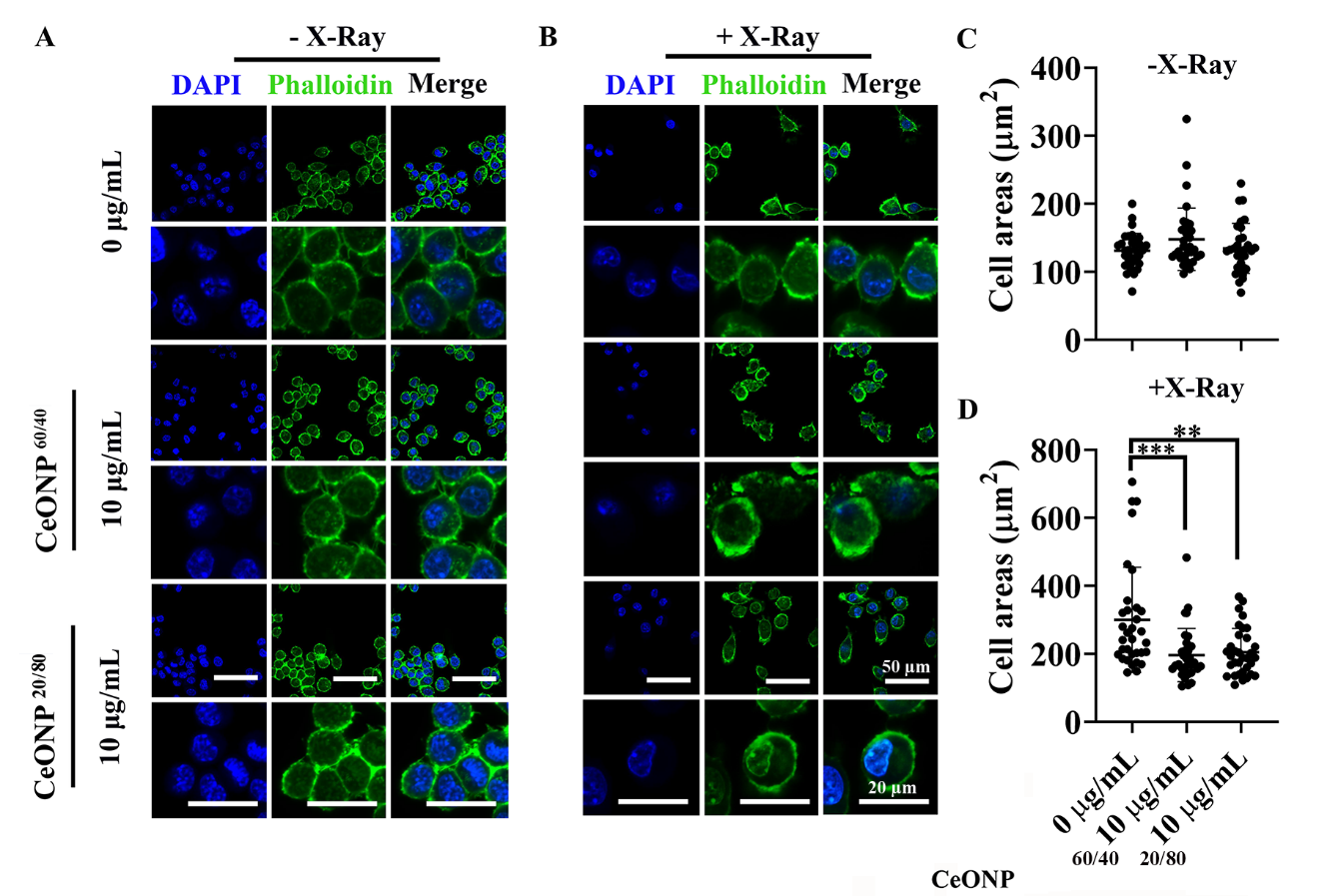


**Supplementary Figure S8. A single exposure to 7 Gy of radiation to RAW264.7 macrophages induces the formation of osteoclast-like, multinucleated giant cells 1-day post-irradiation. CeONP^60/40^ and CeONP^20/80^ treatment both reduce radiation-associated multi-nucleated giant cell formation at this time point.** Macrophage morphology was assessed. Phalloidin (green) was used to stain the actin filaments and DAPI (blue) the cell nuclei. [A-B] Representative high magnification confocal images of RAW264.7 cells after mock-exposure (A) and X-ray exposure (B) 1-day post-irradiation and following 24h pre-treatment with 10 μg/mL of CeONP^60/40^ or CeONP^20/80^. [C-D] Quantification of cell area (μm^2^) in each group. In the absence of irradiation, no changes in cell size were measured when each group was compared. Following exposure to radiation, cells in the untreated (0 μg/mL), control group displayed a significant increase in size. Giant cells are considered a hallmark indicator of chronic inflammatory conditions able to promote bone resorption. Treatment with 10 μg/mL of CeONP^60/40^ or CeONP^20/80^ maintained cell size when compared with control cells and following IR-induced damage. Experiments were carried out in triplicate. All values are given as the mean ± SD. ***p* < 0.01, ****p* < 0.001.

**Supplementary Figure S9**

**
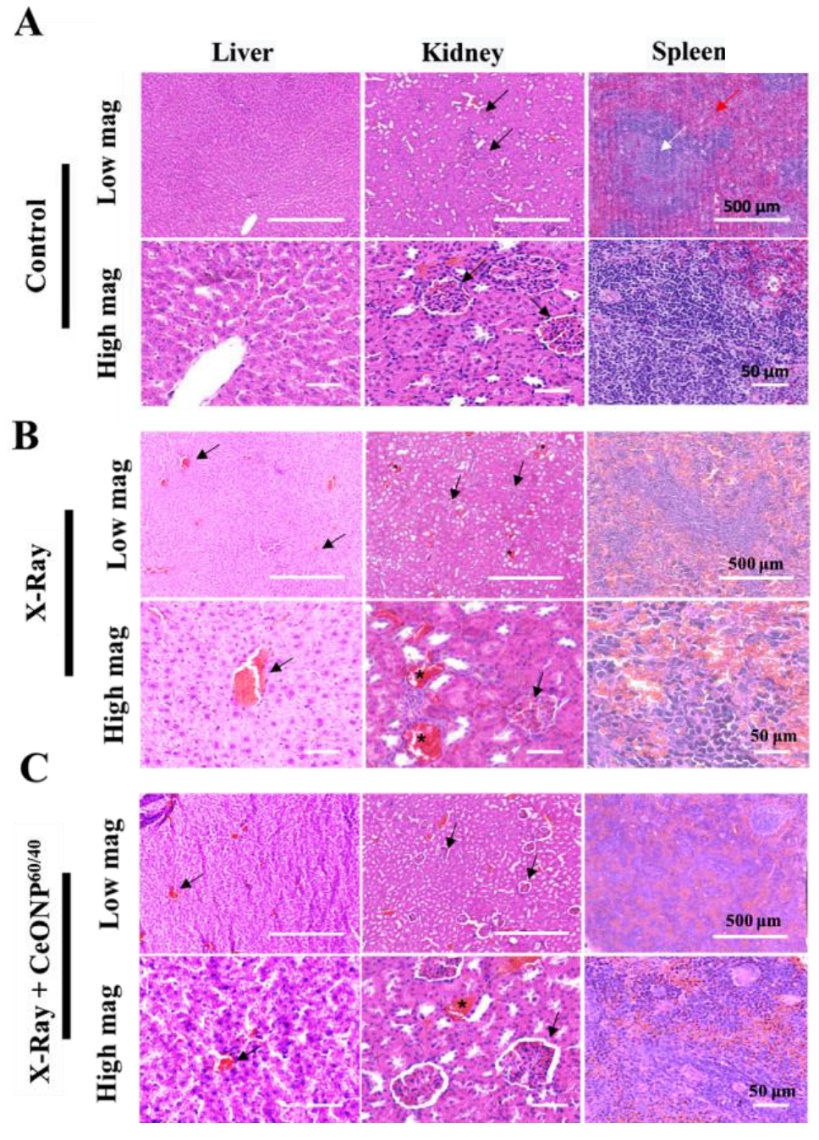
**

**Supplementary Figure S9. Histopathological examination of the liver, kidney and spleen using light microscopy (day 14).** Representative micrographs of the liver, kidney, and spleen in [A] non-irradiated healthy control animals, [B] irradiation-exposed animals (without CeONP treatment). Qualitative histoanalysis of liver tissue in healthy animals, showed a normal distribution of hepatocytes with clear visible nuclei. Radiation-induced hepatopathy was observed as indicated by an increased number of congested terminal hepatic venules (arrows). Radiation-induced nephropathy was also observed as indicated by enlarged glomeruli and dilated renal tubules (arrows highlight glomerulus) and debris accumulation in the tubular lumen (asterisk) with vascular congestion in tubules and glomeruli. Examination of the spleen following exposure to radiation appeared to cause the disappearance of white pulp and the boundaries of the white (lymphocytes) and red (erythrocytes) pulps were vague. Separation within the meshwork of reticular fibers was also evident in this group of animals. In contrast, and in the healthy spleen, the tissue presents with well-defined red pulp (red arrow) and abundant white pulp (white arrow). This is indicated by fewer congested terminal hepatic venules, reduced vascular congestion in tubules and glomeruli in the kidney, and well-defined white and red pulp regions with well-preserved areas of white pulp in the spleen.

**
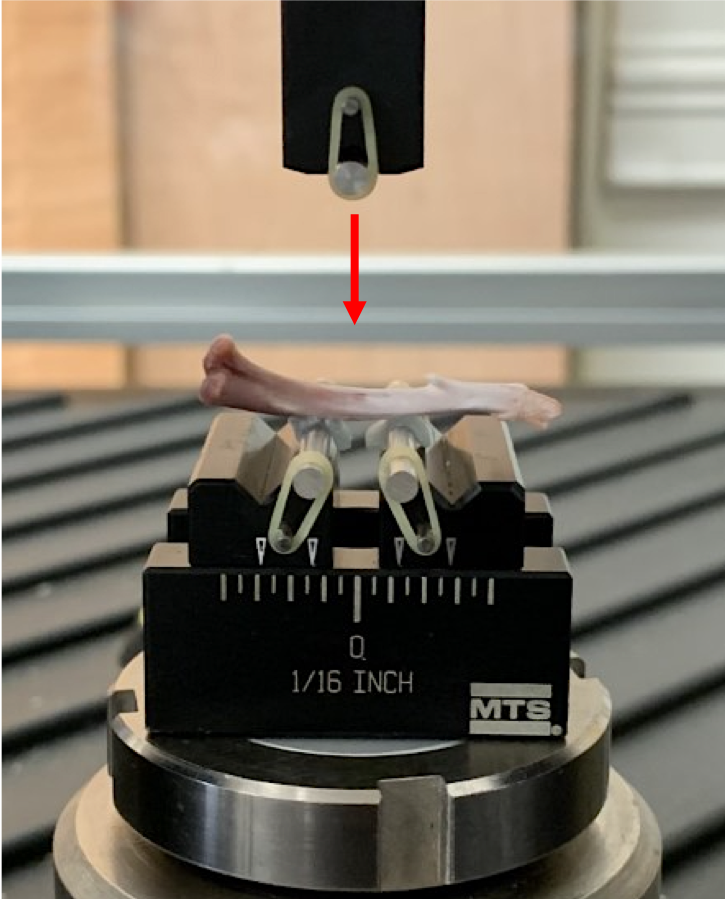
Supplementary Figure S10**

**Supplementary Figure S10. The 3-point bending test fixture.** A vertical force (red arrow) was applied to the mid-shaft of fresh-frozen tibiae hydrated in PBS. Each tibia was obtained on day-7, and loaded to failure using a 3 mm diameter loading roller at a displacement rate of 0.02 mm/s and span length of 8mm using a universal testing machine (Criterion^®^ 43, MTS, Minnesota, USA). Prior to testing, tibiae were positioned horizontally and in the anterior-posterior plane. Based on the recorded load deformation curves and the morphological and biomechanical parameters obtained following microCT (*n*=3), this data was used to determine ultimate stress and fracture stress.

**Supplementary Figure S11**


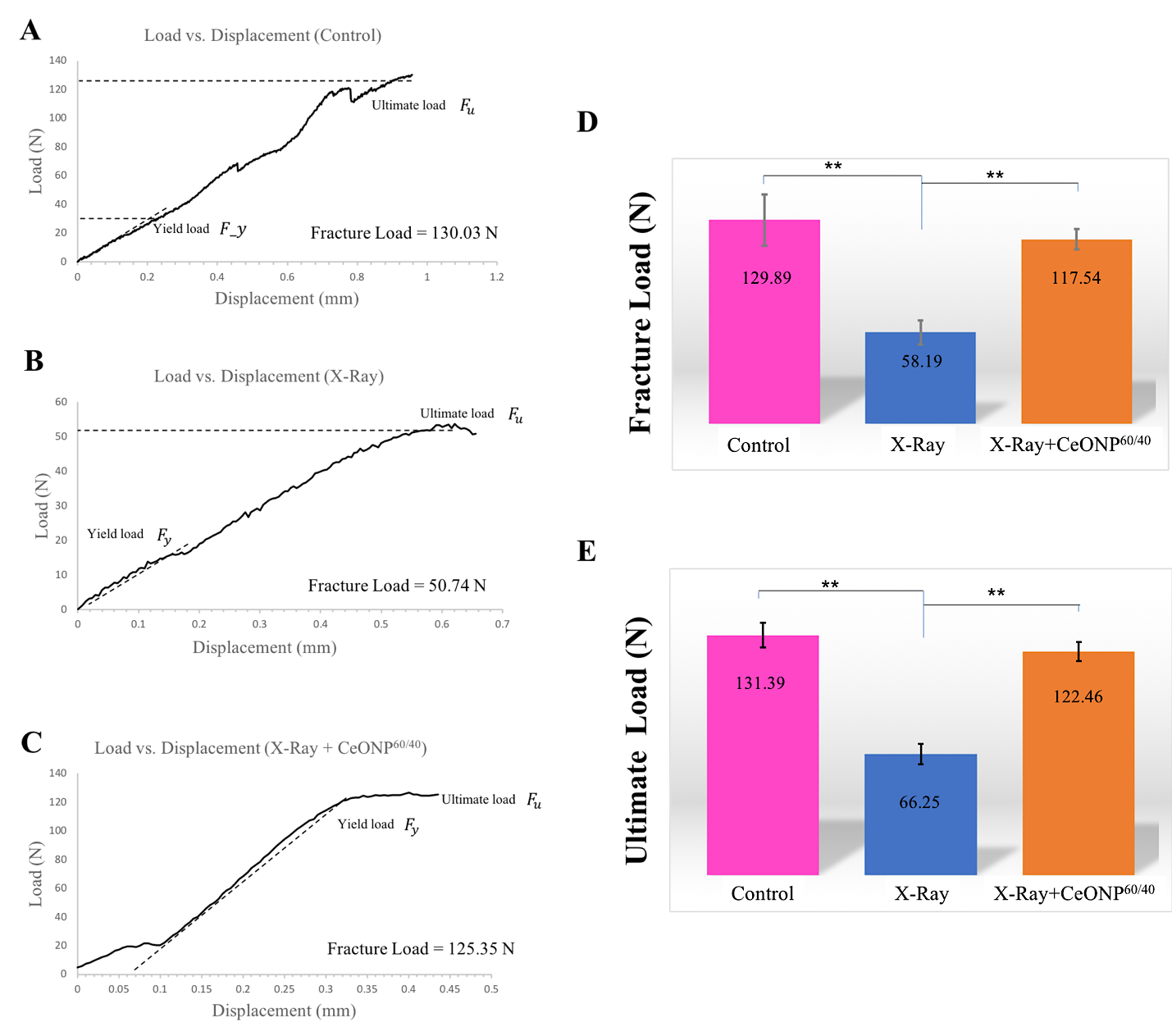


**Supplementary Figure S11.** Representative load-displacement curves of [A] healthy control animals (mean 129.89 ± 16.25N), [B] X-Ray only (mean 58.19 ± 7.60N), and [C] X-Ray + CeONP^60/40^ (mean 117.54 ± 6.44N). [D] fracture load (N) and [E] ultimate load (N) in each of the groups (*n*=6). The total exposure of 24 Gy of radiation to the hind limbs of 9-week old, male SAS resulted in a significant decrease in the amount of load required to break the bone. This indicates the bone fractures when under lower loads and when compared with animals in the healthy control group. Animals receiving CeONP^60/40^ *i.v.* treatment at a dose of 4mg/kg demonstrated higher fracture and ultimate loads suggesting the nanozyme contributed to maintaining bone strength similar to healthy non-irradiated animals.

**Supplementary Table S1**

| **Group** | **No.** | **Outer diameter (mm)** | **Outer radius (mm)** | **Inner radius (mm)** | **Length (mm)** | **Moment of Inertia (mm^4^)** |
| --- | --- | --- | --- | --- | --- | --- |
| **Control** | 1 | 3.10 | 1.55 | 0.88 | 35.45 | 4.06 |
|  | 2 | 3.05 | 1.53 | 0.87 | 36.30 | 3.81 |
|  | 3 | 3.04 | 1.52 | 0.86 | 36.20 | 3.80 |
|  | 4 | 3.31 | 1.65 | 0.93 | 36.36 | 5.28 |
|  | 5 | 3.23 | 1.61 | 0.91 | 35.99 | 4.79 |
|  | 6 | 3.01 | 1.50 | 0.85 | 36.49 | 3.61 |
|  |  |  |  |  |  |  |
| **X-Ray** | 1 | 3.26 | 1.63 | 0.92 | 42.96 | 4.97 |
|  | 2 | 3.29 | 1.65 | 0.93 | 36.05 | 5.16 |
|  | 3 | 3.07 | 1.54 | 0.87 | 35.53 | 3.91 |
|  | 4 | 2.70 | 1.35 | 0.76 | 32.72 | 2.33 |
|  | 5 | 2.64 | 1.32 | 0.74 | 34.02 | 2.13 |
|  | 6 | 2.91 | 1.45 | 0.82 | 33.44 | 3.15 |
|  |  |  |  |  |  |  |
| **X-Ray + CeONP^60/40^** | 1 | 3.02 | 1.51 | 0.86 | 33.50 | 3.66 |
|  | 2 | 3.08 | 1.54 | 0.87 | 34.31 | 3.96 |
|  | 3 | 3.16 | 1.58 | 0.90 | 35.41 | 4.39 |
|  | 4 | 3.17 | 1.58 | 0.89 | 33.61 | 4.44 |
|  | 5 | 3.09 | 1.54 | 0.87 | 36.86 | 4.01 |
|  | 6 | 2.92 | 1.46 | 0.82 | 35.08 | 3.19 |

**Supplementary Table S1.** The biomechanical parameters (outer diameter, outer radius, inner radius, length and moment of inertia) of six tibiae (*n*=6) were determined using microCT. The mean in each group (under a stress of 1000000 Pa) was used to calculate fracture stress and ultimate stress from the load displacement curves generated by each animal during 3-point bending analysis.
